# Supplementary material for: HBV X protein regulates cancer stemness and tumor invasiveness through SENP1 in hepatocellular carcinoma
Source: JHEP Rep. 2025 Oct 8;8(1):101620. doi: 10.1016/j.jhepr.2025.101620 (PMC12721047; doi:10.1016/j.jhepr.2025.101620)
Supplement: Multimedia component 1 [file mmc1.pdf]

HBV X protein regulates cancer stemness and tumor invasiveness through SENP1 in hepatocellular carcinoma

Yu-Chih Wu, Yen-Chiao Huang, Yung-Che Kuo, Mai-Huong Thi Ngo, Kam-Fai Lee, Yen-Tseng Sung, Hsiao-Feng Wang, Shin-Lian Doong, Liang-Mou Kuo, Te-Sheng Chang, Yen-Hua Huang

Table of contents

Supplementary materials and methods..2

Supplementary tables..10

Supplementary figures..13

Supplementary references...21

Supplementary materials and methods

Plasmid construction, short hairpin RNA, and lentiviral transduction

The HBx-IRES-GFP lentiviral vector, which encodes the full-length HBx protein, was reconstructed from the pRT-HBx-GFP plasmid, which was kindly provided by Dr. Shin-Lian Doong (1). The HA-SENP1-IRES-tRFP lentiviral vector was generated in a previous study (2). The LentimiRa-hsa-miR-145-5p Vector (Cat. No. mh40185) for miR-145 overexpression was purchased from Applied Biological Materials Inc. in Canada. The plasmids encoding short hairpin RNAs for SENP1 (shSENP1#1, TRCN0000004395; shSENP1#2, TRCN0000004396), OCT4 (shOCT4#1, TRCN00004879; shOCT4#2, TRCN00004881), and shControl. (shCtrl., TRCN0000072224) were purchased from the National RNAi Core Facility at Academia Sinica in Taiwan. For viral production, the packaging pCMVΔR8.91 plasmid and the envelope VSV-G pMD.G plasmid were cotransfected with the shRNA plasmids into HEK293T cells by using the Turbofect transfection reagent according to the manufacturer's instructions (Thermo Fisher Scientific, USA). After 12-h transfection, the cells were washed with phosphate-buffered saline (PBS), and the cells were then cultured in 10 mL of medium containing 10% FBS. The virus particles were collected at 48 and 72 h post-transfection, filtered through 0.45-μm filters, and concentrated by ultracentrifugation at 140,000 ×*fg* at 4°C for 2.5 h. The cells were infected with the lentivirus in the presence of 8 μg/mL polybrene (Sigma-Aldrich, USA).

Database analysis

For data mining, the gene expression data of the peri-tumor and tumor tissues were downloaded from the Gene Expression Omnibus (GEO) website (GSE76427) (3). The gene set enrichment algorithm (GSEA) was implemented using MSigDB. Moreover, 115 patients with HCC from the GSE76427 dataset were divided into two groups based on their median SENP1 expression level (i.e., SENP1_High and SENP1_Low). Then, the raw data from the GSE76427 dataset were analyzed through GSEA in

reference to the HCC recurrence signature (WOO_LIVER_CANCER_RECURRENCE_UP [M12602] and WOO_LIVER_CANCER_RECURRENCE_DN [M9911]) and embryonic stem cell (ESC) signature (WONG_EMBRYONIC_STEM_CELL_CORE [M7079]) gene sets (3-5). The WOO_LIVER_CANCER_RECURRENCE_UP and WOO_LIVER_CANCER_RECURRENCE_DN gene sets contain the genes positively and negatively correlated with HCC recurrence. The WONG_EMBRYONIC_STEM_CELL_CORE gene set contains the genes coordinately upregulated in a compendium of human ESCs.

RNA isolation and real-time quantitative polymerase chain reaction

Total RNA was isolated from the cell lines and frozen HCC tissues and was subjected to real-time quantitative polymerase chain reaction (qPCR). Specifically, for the cell lines, total RNA was extracted using the RNeasy Micro Kit (Qiagen, USA) according to the manufacturer’s instructions. Frozen tissues were homogenized in liquid N_2 and lysed in RNA extraction buffer. Moreover, 3 μg of total RNA was used to synthesize complementary DNA (cDNA) with a random primer (Thermo Fisher Scientific), and in a final volume of 20 μL, cDNA synthesis was performed using Superscript III reverse transcriptase (Thermo Fisher Scientific) at 42°C for 50 min according to the manufacturer’s instructions. For qPCR amplification, the Fast SYBR Green Master Mix (Thermo Fisher Scientific) was utilized. The primers used in the study are listed in Table S1. Specifically, for the quantification of miR-145 expression, small RNA was extracted from HepG2 cells using the NucleoSpin miRNA kit (Takara Bio, Japan). The cDNA synthesis was conducted using the Mir-X miRNA First Strand Synthesis kit (Takara Bio), adhering to the manufacturer’s guidelines. Subsequent qPCR was performed using the Mir-X™ miRNA qRT-PCR TB Green kit (Takara Bio) with a miR-145-specific primer (5’-GTCCAGTTTTCCCAGGAATCCCT-3’) and the mRQ 3' universal primer. Each reaction was conducted in triplicate, and miR-145 expression levels were normalized to U6 snRNA using the ΔΔCt method.

Western blot analysis

Total protein was extracted using RIPA lysis buffer supplemented with a protease inhibitor cocktail (Roche Diagnostics). Protein concentration was determined using a BCA protein quantification kit (Pierce, USA). For the Western blot analysis, 20 μg of total protein was denatured by boiling in Laemmli buffer, separated by 10% sodium dodecyl sulfate-polyacrylamide gel electrophoresis (SDS-PAGE), and subsequently transferred to a polyvinylidene difluoride (PVDF) membrane. The PVDF membranes were blocked with 5% skim milk in Tris-buffered saline containing 0.05% Tween-20 (TBST) for 1 hour at room temperature. Membranes were then incubated with primary antibodies overnight at 4°C. A detailed list of primary antibodies used is provided in Table S2. Following primary antibody incubation, the membranes were incubated with horseradish peroxidase (HRP)-conjugated goat anti-rabbit or rabbit anti-mouse IgG secondary antibodies for 1 hour at room temperature. After four washes with TBST, immunoreactive bands were visualized using the Immobilon Western blotting kit (EMD Millipore, Germany).

Tissue microarray development and immunohistochemical staining

Matched pairs of paraffin-embedded primary HCC tissue samples and adjacent liver tissues were used for the construction of a tissue microarray (TMA). Briefly, to obtain representative tissue regions, hematoxylin and eosin-stained sections were prepared from each selected donor block. To produce the TMA block, tissue cylinders (1.5 mm in diameter) were punched from the donor block and were transferred to an 18 × 30-mm paraffin block by using an automatic tissue microarrayer (AutoTiss 1000, EverBio Technology, Canada). The resulting TMA block was cut into 5-μm sections, which were placed on 3-aminopropyltriethoxysilane-coated glass slides by using a sliding microtome (Leica SM 2000 R, Meyer Instruments, USA). Immunohistochemical staining was performed on sections obtained from TMA and mouse tissues. Following deparaffinization and rehydration, the slides were washed and blocked with 5% normal horse serum in phosphate-buffered saline (PBS) to reduce non-specific binding. The slides were then incubated with the primary antibodies overnight at 4°C. A list

of the primary antibodies used is provided in Table S2. After incubation, the slides were treated with a horseradish peroxidase (HRP)-conjugated secondary antibody. The intensity of the positive signals was visualized using TissueFAXS imaging system and analyzed using TissueQuest analysis software (TissueGnostics).

Immunocytochemistry staining

Cells were fixed in 4% paraformaldehyde at room temperature for 30 min. After fixation, cells were rinsed two times with PBS at room temperature for 30 min and blocked with bovine serum albumin (BSA; 50 mg/mL) and 0.5% triton X-100 in PBS for 1 h at room temperature. Cells were then incubated with the anti-SENP1 antibody (sc-271360, Santa Cruz Biotechnology) or anti-PIN1 antibody (sc-46660, Santa Cruz Biotechnology), and labeling with the primary antibody was detected using Cy3-conjugated secondary antibodies (Jackson ImmunoResearch, Bar Harbor, ME, USA). The nuclei of all cells were counterstained with 4',6-diamidino-2-phenylindole (DAPI, Sigma-Aldrich). All cells were covered with an anti-fading reagent (Vector Laboratories, Burlingame, CA, USA) and were analyzed under a fluorescence microscope.

Flow cytometry analysis

HepG2 cells, GFP-HepG2 cells and HBx-GFP-HepG2 cells were harvested, washed twice with phosphate-buffered saline (PBS), and resuspended in PBS containing 1% bovine serum albumin (BSA). The cells were then incubated with the PE-conjugated anti-CD133 antibody (BD bioscience, USA) for 30 minutes at 4°C in the dark. Following incubation, the cells were washed twice with PBS to remove unbound antibody. Flow cytometry analysis was performed using the BD FACSVerse flow cytometer. Data acquisition and analysis were conducted using BD FACSDiva software (BD Biosciences).

Wound-closure and transwell migration assays

For the wound-closure assay, cells were added to 60-mm plates at equivalent cell densities. A

micropipette tip was used to scratch a wound in each cell monolayer. The plates were washed with medium to remove the detached cells. Subsequently, the adherent cells were incubated in 5% CO_2 humidified atmosphere at 37°C for 12-24 h. Digital images of the scratch-wound area were acquired at each time point, and the area of the gap was measured. For the transwell assay, cells (2×10^5) were seeded into a Matrigel-coated transwell insert (8-μm pore size, Corning Costar, USA) for 24 h. Uninvaded cells were removed with a cotton swab, and cells that had passed through the lower membrane of the transwell insert were fixed, stained with a crystal violet solution, and observed under an inverted microscope.

Tumor sphere formation assay

Cells (2×10^3) were seeded into a 6-well ultra-low attachment plate (Corning Costar), and cells were maintained in serum-free DMEM-F12 supplemented with 20 ng/mL epidermal growth factor (EGF; PeproTech, USA), 25 ng/mL basic fibroblast growth factor (bFGF; PeproTech), and B27 (Thermo Fisher Scientific) for 14 days for primary tumor sphere formation. The primary spheres were dissociated after 7 days, gently trypsinized, counted and then re-seeded for secondary tumor sphere formation. Tumor spheres were observed and counted under a light microscope. Secondary tumor spheres were observed under a light microscope, and the number of spheres with a diameter greater than 100 μm was counted. The experiment was conducted in triplicate, and the average number of tumor spheres was calculated across the replicates.

Dual luciferase assay

The SENP1-promoter luciferase plasmids, in which the SENP1 promoter was fused with firefly luciferase reporter gene, were cotransfected with the control Renilla luciferase plasmid (pRL-TK) into HepG2 cells (6). Following cotransfection, HepG2 cells were lysed using the cell lysis buffer provided with the Dual Luciferase Reporter 1000 Assay System (Promega, USA), and relative luciferase activity was assessed using a luminometer, Relative luciferase activity is used to represent the ratio of firefly

luciferase activity to Renilla luciferase activity.

Targeting 3'UTR luciferase reporter assay

The SENP1 3'- untranslated region (UTR) sequence, which contains a predicted miR-145-5p binding site, was cloned into a pMirTarget 3'-UTR assay vector (Origene Technologies, Inc., USA) following digestion with the restriction enzymes EcoRI and XbaI. HepG2 cells were infected with the LentimiRa-hsa-miR-145-5p lentivirus, and subsequently transfected with the pMirTarget SENP1 3'-UTR luciferase reporter constructs using Lipofectamine™ 3000 transfection reagent (Thermo Fisher Scientific). After 48 hours of incubation, firefly luciferase activity was measured using the Luciferase Reporter Assay System (Promega). The luciferase activity data were normalized to RFP signals and subsequently analyzed with the SpectraMax iD5 Multi-Mode Microplate Reader (Molecular Devices, USA).

Generation of reporter gene cell line

HepG2 cells stably expressing reporter genes were generated by lentiviral vector transduction. The following pLAS3w. Fluc.Ppuro lentiviral vectors from the National RNAi Core Facility at Academia Sinica in Taiwan were used. Cells were infected with lentivirus to express firefly luciferase and carry the puromycin resistance gene, followed by puromycin selection to establish a stable reporter cell line. Using this stable reporter cell line, we overexpressed HBx or silenced SENP1, followed by orthotopic xenograft mouse experiments

Orthotopic xenograft mouse model and* sorafenib treatment *in vivo

Eight-week-old immunodeficient (Nu/Nu) mice were obtained from the National Laboratory Animal Center, Taiwan. Each mouse was anesthetized, and through an transverse incision made in the upper abdomen by using a micro-syringe, each mouse was orthotopically inoculated in the left hepatic lobe. For tumor growth monitoring, tumors were imaged by IVIS Lumina III XRMS (PerkinElmer Inc.,

USA). Before imaging, 100 µl of 30 mg/mL D-luciferin (Bioxynth, UK) dissolved in DPBS was intraperitoneally injected into each mouse, and 15 min later, the mice were anesthetized with isoflurane and imaged by the IVIS imaging system. After 8 weeks, the mice were sacrificed, and their livers and lungs were dissected, fixed with phosphate-buffered neutral formalin, and prepared for standard histological examination. The number of lung metastatic tumors in mice was determined using tissue sections stained with hematoxylin and eosin (HE). These sections were observed under a microscope, and the quantity of lung metastatic tumor area was counted. To evaluate the therapeutic response to sorafenib, mice in four experimental groups (GFP-shCtrl., GFP-shSENP1, HBx-shCtrl., HBx-shSENP1) were administered sorafenib intraperitoneally at a dose of 30 mg/kg, twice per week, beginning at week 8 post-cell inoculation. Tumor progression was monitored weekly using bioluminescence imaging (IVIS Lumina III XRMS, PerkinElmer Inc., USA). Bioluminescence signals (photons/second) obtained at week 8 served as the baseline and were normalized to 100% for subsequent longitudinal comparisons. The animal study protocol was approved by the Institutional Animal Care and Use Committee at Taipei Medical University, Taipei, Taiwan (Approval number: LAC-2017-0022, LAC-2025-0040).

Dose-response profiles of sorafenib in HCC cell lines

Cells were seeded at a density of 5,000 cells per well in 96-well plates and treated with sorafenib (Cell Signaling Technology, USA), SENP1-IN-3 (MedChemExpress, USA), or their combination for 48 hours. Cell viability was assessed using the WST-1 assay.

Statistical analysis

Data are presented as mean ± standard deviation (SD), as appropriate. The statistical differences in the means were assessed using the paired Student's *t* test, Mann-Whitney test, or Spearman's correlation analysis. Kaplan-Meier analysis was used to examine overall survival (OS) and disease-free survival (DFS), and the Peto-Prentice test was used to determine OS and DFS. $P < 0.05$ indicated statistical

significance. GraphPad Prism 9 software for Windows was used for statistical analysis.

Supplementary tables

Table S1. List of real-time quantitative PCR primers

Gene	Accession	Forward Primers	Reverse Primers
SENP1	NM_001267594	5’-TGGCCAGAGTGCAAATGG-3’	5’-TCGGCTGTTTCTTGATTTTTGTAA-3’
OCT4	NM_002701	5’-CAACTCCGATGGGGCCT-3’	5’-CTTCAGGAGCTTGGCAAATTG-3’
SNAIL	NM_001078353	5’-CTTCCAGCAGCCCTACGAC-3’	5’-CGGTGGGGTTGAGGATCT-3’
TWIST	NM_001165012	5′-TCTCGGTCTGGAGGATGGAG-3′	5′-GTTATCCAGCTCCAGAGTCT-3′
β-2M	NM_004048	5’-GATGAGTATGCCTGCCGTGTG-3’	5’-CAATCCAAATGCGGCATCT-3’

Table S2. List of antibodies

Protein	Assay	Cat. No.	Supplier	Origin	Dilution	Incubation Period
SENP1	IHC	NBP1-89553	Novus	rabbit	1:100	overnight, 4°C
OCT4	IHC	sc-5279	Santa Cruz Biotech	mouse	1:100	overnight, 4°C
CD133	IHC	ab19898	Abcam	rabbit	1:200	overnight, 4°C
N-Cadherin	IHC	610920	BD bioscience	mouse	1:200	overnight, 4°C
E-Cadherin	IHC	610182	BD bioscience	mouse	1:200	overnight, 4°C
PIN1	IHC	sc-15340	Santa Cruz Biotech	rabbit	1:100	overnight, 4°C
SENP1	WB	sc-271360	Santa Cruz Biotech	mouse	1:1000	overnight, 4°C
OCT4	WB	sc-5279	Santa Cruz Biotech	mouse	1:500	overnight, 4°C
N-Cadherin	WB	610920	BD bioscience	mouse	1:1000	overnight, 4°C
E-Cadherin	WB	610182	BD bioscience	mouse	1:1000	overnight, 4°C
SNAIL	WB	GTX125918	GeneTex	rabbit	1:1000	overnight, 4°C
TWIST	WB	GTX127310	GeneTex	rabbit	1:1000	overnight, 4°C
HBx	WB	sc-71239	Santa Cruz Biotech	mouse	1:1000	overnight, 4°C
CD133	WB	#64326	Cell Signaling	rabbit	1:2000	overnight, 4°C
PIN1	WB	sc-15340	Santa Cruz Biotech	mouse	1:2000	overnight, 4°C
CyclinD1	WB	2261-1	Epitomic	rabbit	1:20000	overnight, 4°C
p-STAT3	WB	#9145	Cell Signaling	rabbit	1:2000	overnight, 4°C
STAT3	WB	#9132	Cell Signaling	rabbit	1:2000	overnight, 4°C
IGF-1β	WB	sc-713	Santa Cruz Biotech	rabbit	1:1000	overnight, 4°C
β-ACTIN	WB	A5441	Sigma-Aldrich	mouse	1:10000	overnight, 4°C
CD133	FC	566593	BD bioscience	mouse	1:100	30 minutes, 4°C
SENP1	ICC	NBP1-89553	Novus	rabbit	1:100	overnight, 4°C
PIN1	ICC	sc-15340	Santa Cruz Biotech	rabbit	1:100	overnight, 4°C

IHC, immunohistochemistry; WB, Western blot; FC, flow cytometry;
ICC, immunocytochemistry

Table.S3 Number of primary tumors, intrahepatic metastasis and pulmonary metastasis in orthotopic xenograft mice.

Group	Exp. Condition		Primary tumor	Intrahepatic metastasis	Pulmonary metastasis
**1**	GFP	shCtrl.	3/4	1/4	1/4
**2**		shSENP1	1/4	0/4	0/4
**3**	HBx	shCtrl.	4/4	4/4	3/4
**4**		shSENP1	2/3	1/3	0/3

Supplementary figures

Fig. S1

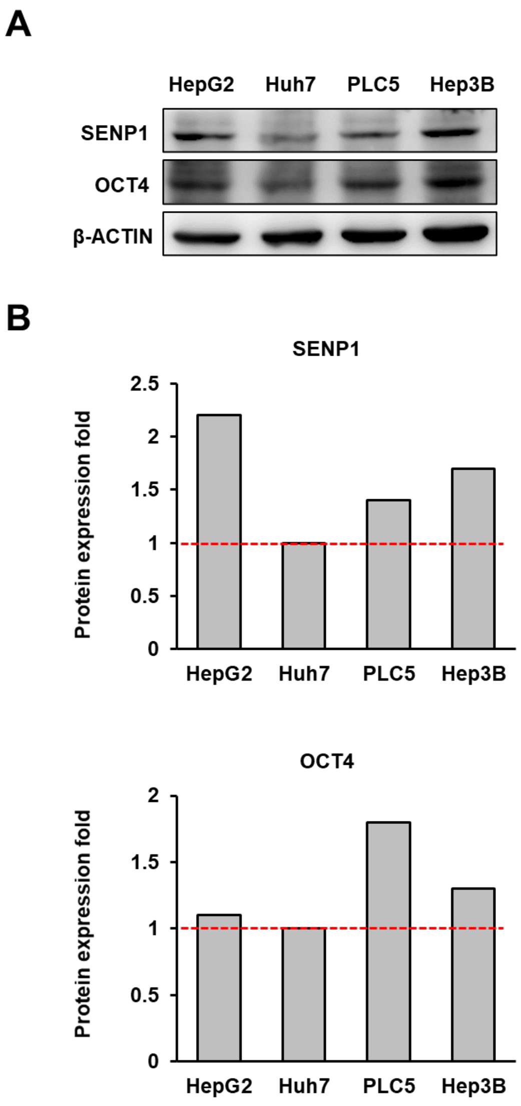

Fig. S1. SENP1 and OCT4 protein levels in various commercialized HCC cell lines.

A, SENP1 and OCT4 protein levels in in various cells were analyzed through Western blotting. **B,** Quantitative data are shown in terms of protein levels.

Fig. S2

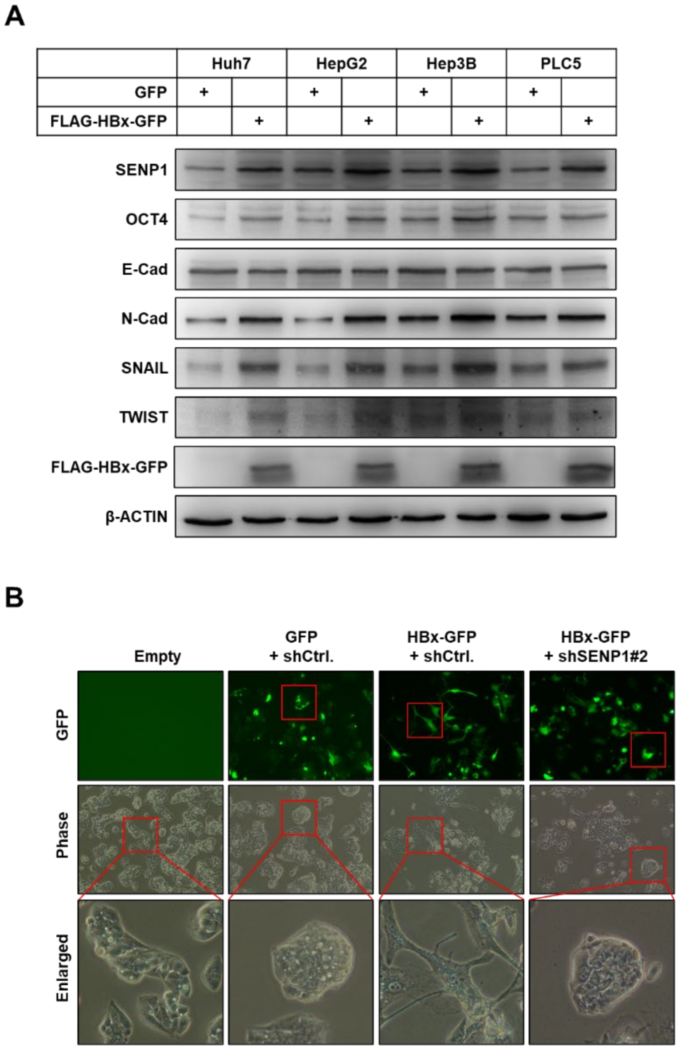

Fig. S2. HBx increases CSC and EMT-associated factors in HCC.

A, Huh7 and HepG2 (HBV^- cell line) and Hep3B and PLC5 (HBV^+ cell lines) were infected the lentivirus carrying the HBx-GFP plasmid or GFP control vector. Relative SENP1, OCT4, E-Cadherin, N-Cadherin, SNAIL, TWIST, and HBx protein levels were examined through Western blotting. **B,** HBx-induced mesenchymal-like cell morphology was changed into epithelial-like cell morphology through SENP1 silencing.

Fig. S3

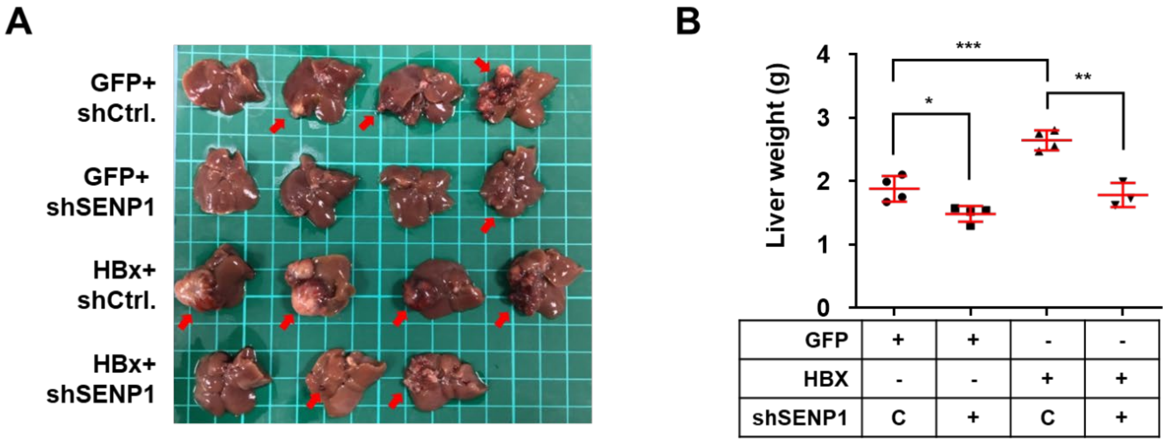

Fig. S3. SENP1 knockdown reduces HBx-induced liver enlargement in orthotopic xenograft model.

A, Representative liver tumor images from four groups of mice: GFP-shCtrl., GFP-shSENP1, HBx-shCtrl., and HBx-shSENP1. Red arrows indicate tumor samples selected for histological analysis, which are presented as representative images in Fig. 6H. **B,** Quantification of liver weights in the indicated groups. *$P < 0.05$, **$P < 0.01$, ***$P < 0.001$, Mann-Whitney test.

Fig. S4

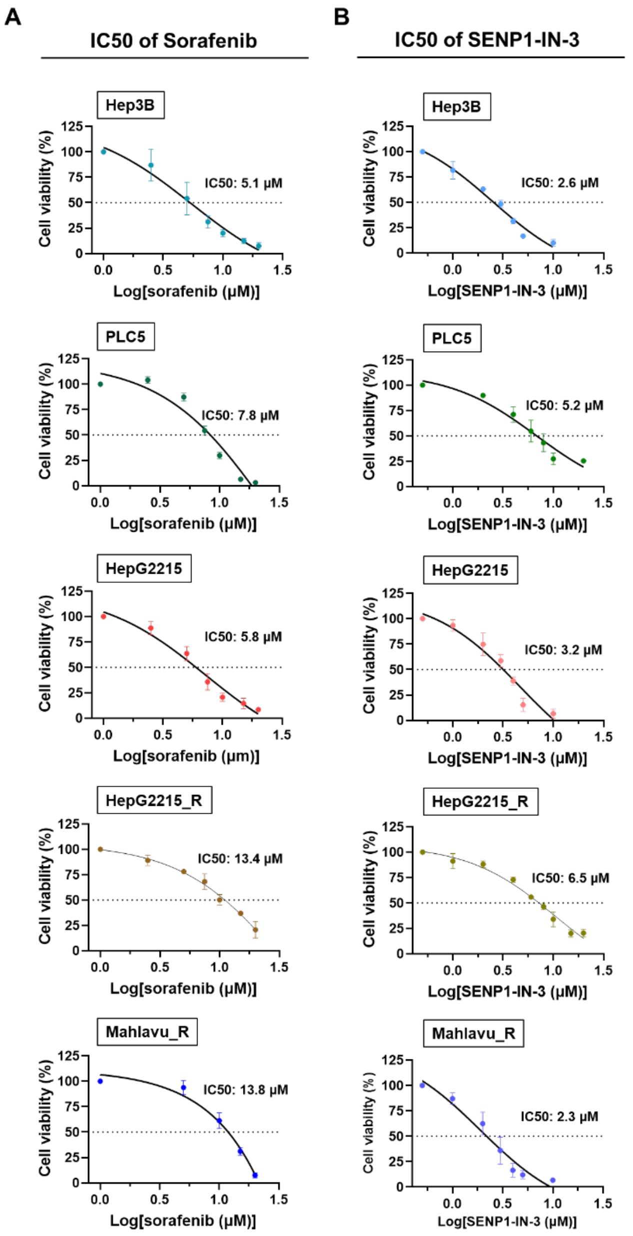

Fig. S4. Dose-response analysis of sorafenib and SENP1-IN-3 in HCC cell lines.

A, IC50 values of sorafenib. **B,** IC50 values of SENP1-IN-3.

Naïve cells (Hep3B, PLC5, and HepG2215) and sorafenib-resistant cells (HepG2215_R and Mahlavu_R) HCC cell lines were treated with increasing concentrations of sorafenib or SENP1-IN-3 for 48 hours. Cell viability was determined using the WST-1 assay. Data represent mean ± SD from more than three independent experiments.

Fig. S5

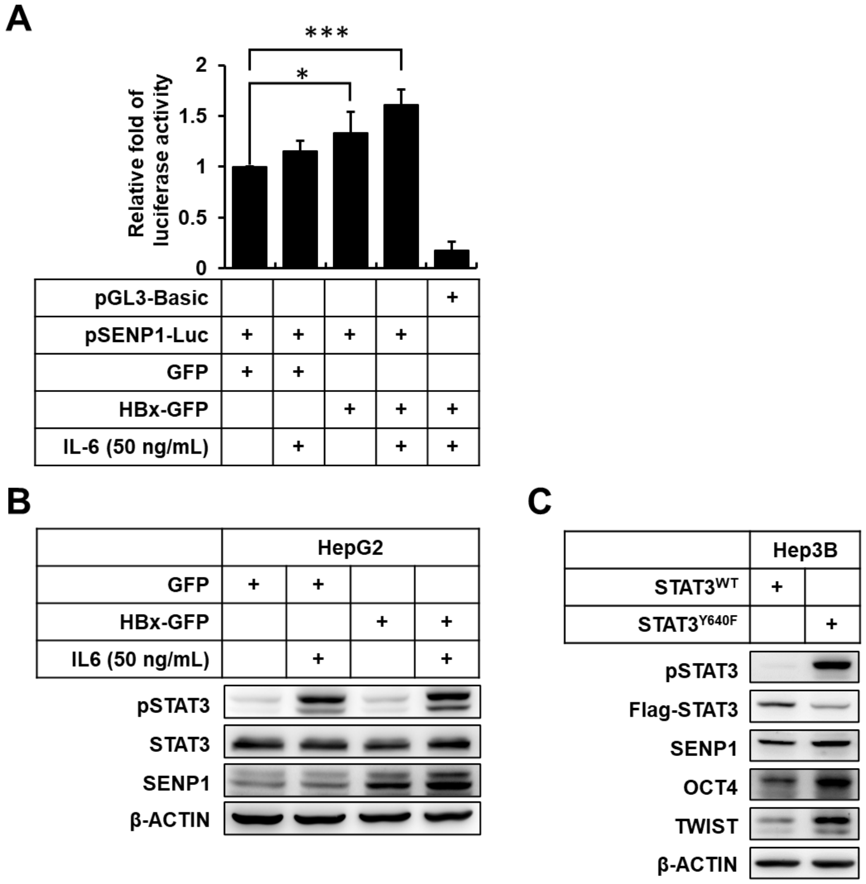

Fig. S5. HBx/IL-6/STAT3 signaling pathway involves SENP1 and OCT4 expression in HBV-related HCC.

A, SENP1 promoter luciferase reporter construct (pSENP1-Luc) or pGL3-empty reporter plasmid was cotransfected with GFP or HBx-GFP plasmids into HepG2 cells. Relative luciferase activity of SENP1 promoter-luciferase HepG2 cells with IL-6 treatment (50 ng/mL) is presented. $*P < 0.05$, $***P < 0.001$, Student's *t* test. **B,** GFP- and HBx-GFP-HepG2 cells treated with IL-6 (50 ng/mL). Relative phosphorylated STAT3 (pSTAT3), total STAT3 (STAT3), SENP1, and HBx protein expression levels detected through Western blotting. **C,** Flag-tagged wild-type STAT3^{WT} expression plasmid and a single mutant STAT3^{Y640F} expression plasmid (a constitutive active STAT3) transfected into Hep3B cells. Phosphorylated STAT3 (pSTAT3), Flag-STAT3, SENP1, OCT4 and TWIST protein levels were detected through Western blotting. β-ACTIN is a loading control.

Fig. S6

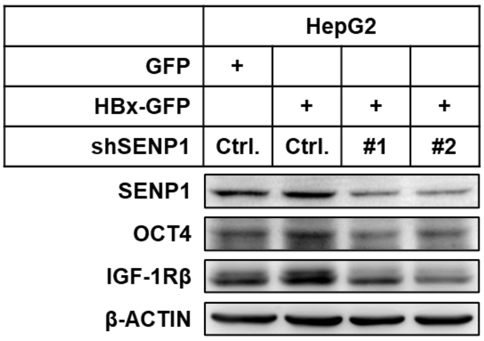

Fig. S6. HBx-induced SENP1 involves IGF-1R and OCT4 expression in HepG2 cells.

SENP1 expression was silenced in GFP- and HBx-GFP-HepG2 cells with shCtrl. and shSENP1 (clone#1 and #2). Relative SENP1, OCT4, IGF-1R β subunit, and β-ACTIN protein levels were examined through Western blotting.

Fig. S7

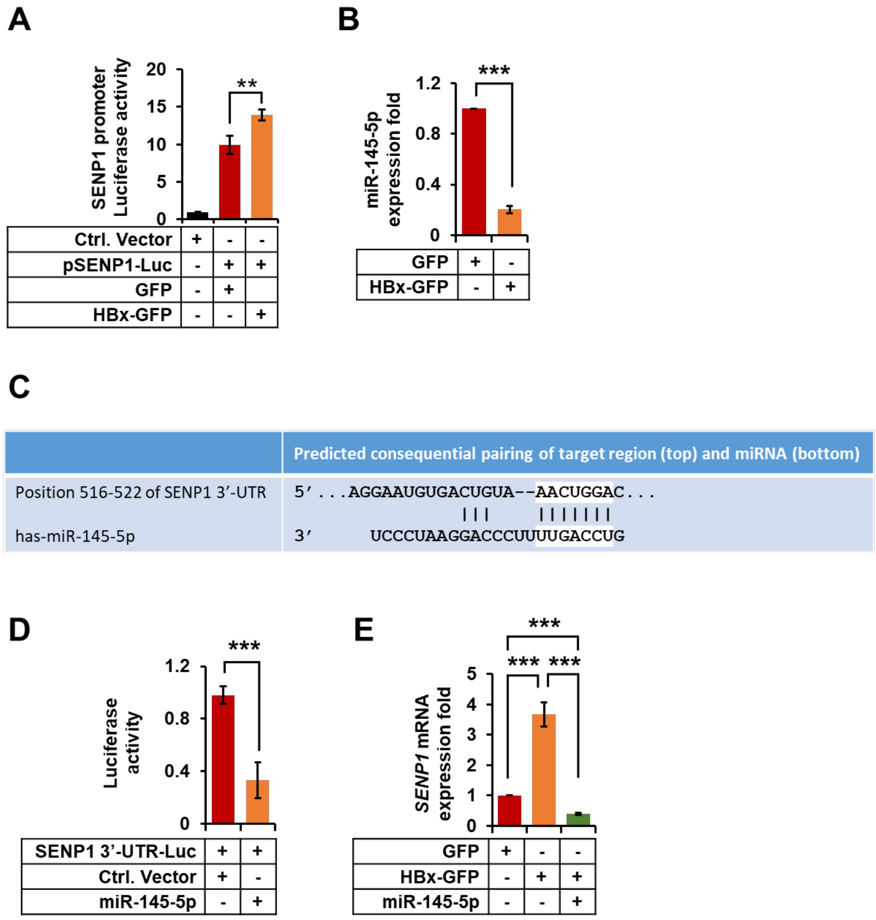

Fig. S7. SENP1 is a direct target of miR-145-5p in HepG2 cells.

A, Luciferase reporter assay of SENP1 promoter activity in HepG2 cells. **B,** MiR-145 expression levels in GFP- or HBx-overexpressing HepG2 cells were analyzed through qPCR. **C,** Sequence alignment of miR-145-5p with SENP1 3'-UTR. **D,** HepG2 cells were infected with lentivirus carrying miR-145-5p alongside pMirTarget SENP1 3 ˊ -UTR luciferase reporter constructs for 48 h. Luciferase activity was measured and normalized to the RFP intensity. **E,** HepG2 cells were transduced with different expression plasmids, and *SENP1* mRNA expression levels were analyzed through qPCR. $^{**}P < 0.01$, $^{***}P < 0.001$, paired Student's *t* test.

Fig. S8

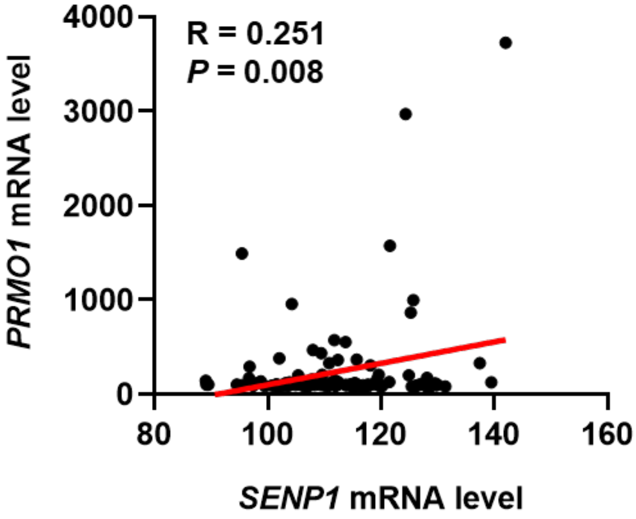

Fig. S8. SENP1 expression positively correlates with CD133 (*PROM1*) mRNA levels in HCC tissue samples.

A significant positive correlation between SENP1 and CD133 (*PROM1*) mRNA expression was observed in HCC specimens. The data were obtained from the GEO database (GSE76427). Pearson's correlation test.

Supplementary references

1. Shih WL, Kuo ML, Chuang SE, Cheng AL, Doong SL. Hepatitis B virus X protein inhibits transforming growth factor-beta -induced apoptosis through the activation of phosphatidylinositol 3-kinase pathway. J Biol Chem. 2000;275(33):25858-64.
2. Wu YC, Ling TY, Lu SH, Kuo HC, Ho HN, Yeh SD, et al. Chemotherapeutic sensitivity of testicular germ cell tumors under hypoxic conditions is negatively regulated by SENP1-controlled sumoylation of OCT4. Cancer Res. 2012;72(19):4963-73.
3. Grinchuk OV, Yenamandra SP, Iyer R, Singh M, Lee HK, Lim KH, et al. Tumor-adjacent tissue co-expression profile analysis reveals pro-oncogenic ribosomal gene signature for prognosis of resectable hepatocellular carcinoma. Mol Oncol. 2018;12(1):89-113.
4. Wong DJ, Liu H, Ridky TW, Cassarino D, Segal E, Chang HY. Module map of stem cell genes guides creation of epithelial cancer stem cells. Cell Stem Cell. 2008;2(4):333-44.
5. Bhattacharya B, Miura T, Brandenberger R, Mejido J, Luo Y, Yang AX, et al. Gene expression in human embryonic stem cell lines: unique molecular signature. Blood. 2004;103(8):2956-64.
6. Wang C, Tao W, Ni S, Chen Q, Zhao Z, Ma L, et al. Tumor-suppressive microRNA-145 induces growth arrest by targeting SENP1 in human prostate cancer cells. Cancer Sci. 2015;106(4):375-82.
