# Supplementary material for: HBV X protein regulates cancer stemness and tumor invasiveness through SENP1 in hepatocellular carcinoma
Source: JHEP Rep. 2025 Oct 8;8(1):101620. doi: 10.1016/j.jhepr.2025.101620 (PMC12721047; doi:10.1016/j.jhepr.2025.101620)
Supplement: Multimedia component 2 [file mmc2.docx]

**JHEP Reports**

**CTAT methods**

Tables for a “Complete, Transparent, Accurate and Timely account” (CTAT) are now mandatory for all revised submissions. The aim is to enhance the reproducibility of methods.

- Only include the parts relevant to your study
- Refer to the CTAT in the main text as ‘Supplementary CTAT Table’
- Do not add subheadings
- Add as many rows as needed to include all information
- Only include one item per row

**If the CTAT form is not relevant to your study, please outline the reasons why:**

| N/A |
| --- |

- 1. **Antibodies**

| **Name** | **Citation** | **Supplier** | **Cat no.** | **Clone no.** |
| --- | --- | --- | --- | --- |
| SENP1 |  | Novus | NBP1-89553 |  |
| SENP1 |  | Santa Cruz Biotech | sc-271360 |  |
| OCT4 |  | Santa Cruz Biotech | sc-5279 |  |
| N-Cadherin |  | BD bioscience | 610920 |  |
| E-Cadherin |  | BD bioscience | 610182 |  |
| SNAIL |  | GeneTex | GTX125918 |  |
| TWIST |  | GeneTex | GTX127310 |  |
| HBx |  | Santa Cruz Biotech | sc-71239 |  |
| CD133 |  | Cell Signaling | #64326 |  |
| CD133 |  | Abcam | ab19898 |  |
| CD133-PE |  | BD bioscience | 566593 |  |
| PIN1 |  | Santa Cruz Biotech | sc-15340 |  |
| CyclinD1 |  | Epitomic | 2261-1 |  |
| p-STAT3 |  | Cell Signaling | #9145 |  |
| STAT3 |  | Cell Signaling | #9132 |  |
| IGF-1β |  | Santa Cruz Biotech | sc-713 |  |
| β-ACTIN |  | Sigma-Aldrich | A5441 |  |

- 1. **Cell lines**

| **Name** | **Citation** | **Supplier** | **Cat no.** | **Passage no.** | **Authentication test method** |
| --- | --- | --- | --- | --- | --- |
| HepG2 |  | BCRC, Taiwan | RM60025 |  |  |
| Huh7 |  | JCRB, Japan | JCRB0403 |  |  |
| Hep3B |  | BCRC, Taiwan | 60434 |  |  |
| PLC5 |  | BCRC, Taiwan | 60223 |  |  |
| HepG2215 |  | provided by Dr. Jun-Jen Liu (Taipei Medical University, Taipei, Taiwan) |  |  |  |
| Mahlavu |  | provided by Dr. Muh-Hwa Yang (National Yang Ming Chiao Tung University, Taipei, Taiwan) |  |  |  |

- 1. **Organisms**

| **Name** | **Citation** | **Supplier** | **Strain** | **Sex** | **Age** | **Overall n number** |
| --- | --- | --- | --- | --- | --- | --- |
| Nu/Nu mice |  | National Laboratory Animal Center, Taiwan | CAnN.Cg-Foxn1nu/CrlNarl | female | 8 week | 40 |

- 1. **Sequence based reagents**

| **Name** | **Sequence** | **Supplier** |
| --- | --- | --- |
| SENP1-Forward Primer | 5’-TGGCCAGAGTGCAAATGG-3’ | Mission biotech, Taiwan |
| SENP1-Reverse Primer | 5’-TCGGCTGTTTCTTGATTTTTGTAA-3’ | Mission biotech, Taiwan |
| OCT4-Forward Primer | 5’-CAACTCCGATGGGGCCT-3’ | Mission biotech, Taiwan |
| OCT4-Reverse Primer | 5’-CTTCAGGAGCTTGGCAAATTG-3’ | Mission biotech, Taiwan |
| SNAIL-Forward Primer | 5’-Cttccagcagccctacgac-3’ | Mission biotech, Taiwan |
| SNAIL-Reverse Primer | 5’-Cggtggggttgaggatct-3’ | Mission biotech, Taiwan |
| TWIST-Forward Primer | 5′-TCTCGGTCTGGAGGATGGAG-3′ | Mission biotech, Taiwan |
| TWIST-Reverse Primer | 5′-GTTATCCAGCTCCAGAGTCT-3′ | Mission biotech, Taiwan |
| β-2M-Forward Primer | 5’-GATGAGTATGCCTGCCGTGTG-3’ | Mission biotech, Taiwan |
| β-2M-Reverse Primer | 5’-CAATCCAAATGCGGCATCT-3’ | Mission biotech, Taiwan |
| miR-145-specific primer | 5’-GTCCAGTTTTCCCAGGAATCCCT-3’ | Mission biotech, Taiwan |

- 1. **Biological samples**

| **Description** | **Source** | **Identifier** |
| --- | --- | --- |
| HCC tissues | Chang Gung Memorial Hospital (Chiayi, Taiwan) | IRB Approval No. 101-5232B |

- 1. **Deposited data**

| **Name of repository** | **Identifier** | **Link** |
| --- | --- | --- |
|  |  |  |

- 1. **Software**

| **Software name** | **Manufacturer** | **Version** |
| --- | --- | --- |
| GSEA software |  | V4.3.3 |
| GraphPad Prism |  | V9.0.0 |

- 1. **Other (*e.g*. drugs, proteins, vectors etc.)**

| **Name** | **Cat no.** | **Supplier** |
| --- | --- | --- |
| short hairpin RNAs for SENP1#1 (shSENP1#1) | TRCN0000004395 | National RNAi Core Facility at Academia Sinica, Taiwan |
| shSENP1#2 | TRCN0000004396 | National RNAi Core Facility at Academia Sinica, Taiwan |
| shOCT4#1 | TRCN00004879 | National RNAi Core Facility at Academia Sinica, Taiwan |
| shOCT4#2 | TRCN00004881 | National RNAi Core Facility at Academia Sinica, Taiwan |
| shControl | TRCN0000072224 | National RNAi Core Facility at Academia Sinica, Taiwan |

- 1. **Please provide the details of the corresponding methods author for the manuscript:**

| Yen-Hua Huang, PhD,  Department of Biochemistry and Molecular Cell Biology, School of Medicine, Graduate Institute of Medical Sciences, College of Medicine; Research Center for Cell Therapy and Regeneration Medicine, Taipei Medical University, No. 250, Wuxing Street, Taipei 110, Taiwan, Tel: 886-2-27361661 ext. 3150, Email: rita1204@tmu.edu.tw;  Te-Sheng Chang, MD, PhD,  Department of Gastroenterology & Hepatology, Chang Gung Memorial Hospital, Chiayi, Section 8 West Chia-Pu Road, Puzih City, Chiayi County, Taiwan 613, E-mail: cgmh3621@cgmh.org.tw |
| --- |

**2.0 Please confirm for randomised controlled trials all versions of the clinical protocol are included in the submission. These will be published online as supplementary information.**

| N/A |
| --- |
