# Supplementary material for: HBV X protein regulates cancer stemness and tumor invasiveness through SENP1 in hepatocellular carcinoma
Source: JHEP Rep. 2025 Oct 8;8(1):101620. doi: 10.1016/j.jhepr.2025.101620 (PMC12721047; doi:10.1016/j.jhepr.2025.101620)
Supplement: Multimedia component 4 [file mmc4.pdf]

JHEP|Reports

# HBV X protein regulates cancer stemness and tumor invasiveness through SENP1 in hepatocellular carcinoma

## Authors

Yu-Chih Wu, Yen-Chiao Huang, Yung-Che Kuo, …, Liang-Mou Kuo, Te-Sheng Chang, Yen-Hua Huang

## Correspondence

yuchihwu@tmu.edu.tw (Y.-C. Wu), cgmh3621@cgmh.org.tw (T.-S. Chang), rita1204@tmu.edu.tw (Y.-H. Huang).

## Graphical abstract

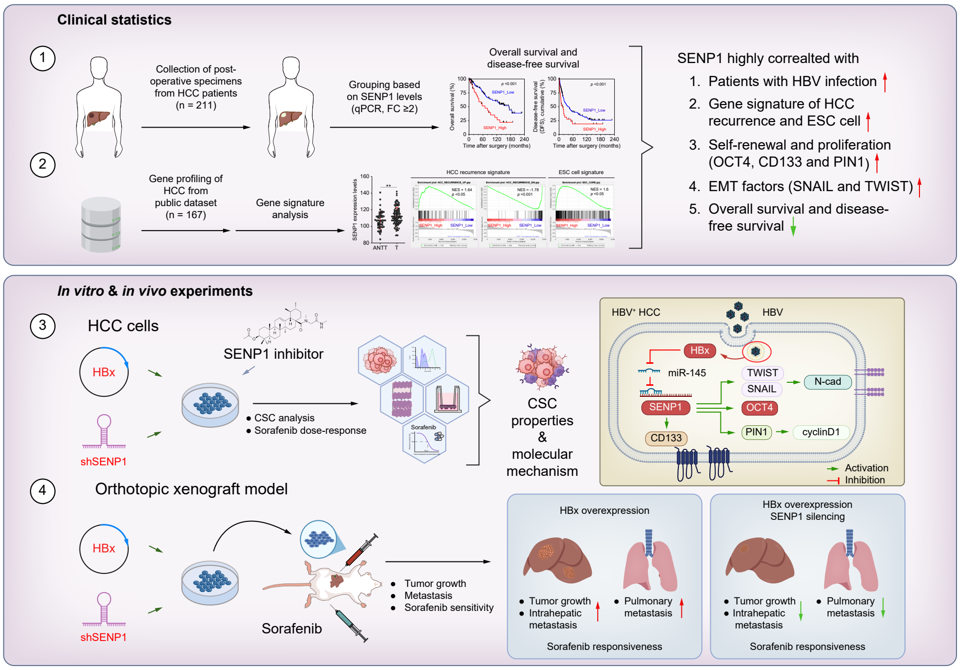


## Highlights:

- Elevated SENP1 is associated with stemness, poor prognosis, and early recurrence, particularly in patients with HBV-related HCC.
- SENP1 regulates CSC-associated properties in HCC, including OCT4, CD133, PIN1, the EMT, and tumor metastasis.
- Suppressing SENP1 significantly enhanced sorafenib sensitivity in HCC both *in vitro* and *in vivo.*
- The HBx-SENP1 pathway regulates cancer stemness-related properties *in vitro* and *in vivo.*

## Impact and implications:

Early recurrence, tumor metastasis, and drug resistance are significant therapeutic challenges in hepatocellular carcinoma (HCC), which are closely associated with cancer stem cell (CSC)-related properties. In this study, we demonstrated that HBx-induced SENP1 expression regulates CSC-related properties and tumor metastasis, particularly in HBV-related HCC, in clinical, *in vitro*, and *in vivo* settings. Findings from this research highlight that HBx-induced SENP1 is crucial for promoting CSC-associated properties in HCC. SENP1 could serve as a novel biomarker of early tumor recurrence and metastasis, especially for HBV-related HCC.

https://doi.org/10.1016/j.jhepr.2025.101620

© 2025 The Author(s). Published by Elsevier B.V. on behalf of European Association for the Study of the Liver (EASL). This is an open access article under the CC BY license (http://creativecommons.org/licenses/by/4.0/). 

# HBV X protein regulates cancer stemness and tumor invasiveness through SENP1 in hepatocellular carcinoma

Yu-Chih Wu[1,2,3], Yen-Chiao Huang[4,5], Yung-Che Kuo[6,7,†], Mai-Huong Thi Ngo[4,†], Kam-Fai Lee[8], Yen-Tseng Sung[4,5], Hsiao-Feng Wang[6], Shin-Lian Doong[9], Liang-Mou Kuo[10], Te-Sheng Chang[11,12,13,*], Yen-Hua Huang[2,4,5,6,14,*]

JHEP Reports **2026**. vol. 8 | 1–14

Check for updates

**Background & Aims:** The niche in hepatocellular carcinoma (HCC) critically influences cancer stem cell (CSC)-associated properties, including stemness, early recurrence, and poor prognoses. HBV infection acts as a niche driver for tumor progression and malignancy. While the HBV X (HBx) protein has been linked to CSC-associated properties, the underlying molecular mechanisms remain unclear.

**Methods:** Paired tumor and peritumor tissues from 211 patients with HCC were analyzed to correlate *SENP1*, *OCT4*, *SNAIL*, *PIN1*, and *TWIST* expression with overall survival (OS) and disease-free survival (DFS) using a Kaplan-Meier survival analysis. Validation was performed using HCC microarray data (n = 167). HBx-SENP1's role in regulating CSC-associated properties was examined *in vitro* (stemness expression, sphere formation, $CD133^+$ cells, migration/invasion, and sorafenib sensitivity) and *in vivo* using an orthotopic xenograft model.

**Results:** Clinically, SENP1 expression was correlated with OCT4, SNAIL, and TWIST ($p$ <0.001), and was associated with poor OS (69.2 *vs.* 172.8 months, $p$ <0.001) and DFS (15.8 *vs.* 39.7 months, $p$ <0.001). SENP1 expression was correlated with gene sets linked to HCC recurrence and embryonic stem cell signatures. In HBV-related HCC, elevated SENP1 (7.8 *vs.* 15.7 months, $p$ = 0.003), OCT4 (7.8 *vs.* 16.7 months, $p$ <0.001), SNAIL (8.6 *vs.* 15.7 months, $p$ = 0.012), and TWIST (8.2 *vs.* 15.5 months, $p$ = 0.028) were linked to early recurrence. Mechanistically, HBx induced CSC-associated properties through SENP1, including sphere formation, $CD133^+$ cells, migration/invasion, and sorafenib resistance. SENP1-knockdown decreased HBx-induced pulmonary metastases and sorafenib refractoriness *in vivo*.

**Conclusions:** HBx-induced SENP1 is critical for CSC properties. SENP1 can serve as a novel biomarker for early recurrence, metastasis, and drug resistance, particularly in HBV-related HCC.

© 2025 The Author(s). Published by Elsevier B.V. on behalf of European Association for the Study of the Liver (EASL). This is an open access article under the CC BY license (http://creativecommons.org/licenses/by/4.0/).

## Introduction

Hepatocellular carcinoma (HCC), the predominant form of primary liver cancer, ranks as the third leading cause of cancer-related mortality worldwide and accounts for approximately 80% of liver cancer cases.[1] Chronic infection with the HBV remains the principal etiological factor, especially in the Asian-Pacific and sub-Saharan African regions, although the incidence of metabolic dysfunction-associated steatotic liver disease (MASLD)-related HCC is rising in Western populations.[2] Despite curative strategies such as surgical resection and local ablation, recurrence rates remain high, with intrahepatic recurrence being most common and pulmonary metastases representing the predominant extrahepatic manifestation.[3–5] Intrahepatic recurrences can arise from occult metastases within 2 years or as *de novo* tumors >2 years after surgery.[6] Preventing postoperative HCC recurrence is crucial for improving prognoses, but effective strategies remain limited despite numerous clinical trials.

A recent study demonstrated that cancer stem cells (CSCs) play an important role in early HCC recurrence, especially in HBV-related HCC.[7] However, the role of HBV in HCC recurrence and CSC-associated properties remains unclear. Numerous studies have shown that the HBV X (HBx) protein plays an important role in HCC development.[8,9] The HBx protein was reported to induce CSC-associated properties, such as upregulation of the OCT4 and NANOG proteins in HCC, although the underlying mechanism remains unclear.[10] Our previous research indicated that patients with HBV-related HCC have shorter disease-free survival (DFS) compared to those with non-HBV/non-HCV, HCV-related, or HBV/HCV-related HCC.[7] We also demonstrated that SUMO/sentrin-specific protease 1 (SENP1) regulates OCT4 stability and

* Corresponding authors. Addresses: Department of Biochemistry and Molecular Cell Biology, School of Medicine, Graduate Institute of Medical Sciences, College of Medicine; TMU Research Center for Cell Therapy and Regeneration Medicine, Taipei Medical University, 250 Wuxing Street, Taipei 11031, Taiwan, Tel.: +886-2-27361661 ext. 3150 (Y.-H. Huang), or Department of Gastroenterology & Hepatology, Chang Gung Memorial Hospital, Chiayi, Section 8 West Chia-Pu Road, Puzih City 613, Chiayi County, Taiwan (T.-S. Chang).
*E-mail addresses:* yuchihwu@tmu.edu.tw (Y.-C. Wu), cgmh3621@cgmh.org.tw (T.-S. Chang), rita1204@tmu.edu.tw (Y.-H. Huang).
† These authors contributed equally to this work.
https://doi.org/10.1016/j.jhepr.2025.101620

activity in embryonal carcinoma cells.[11] Nonetheless, how OCT4 is regulated in HCC remains to be elucidated. Additionally, PIN1, a phosphorylation-dependent prolyl isomerase, is overexpressed in HCC and promotes tumorigenesis by modulating cell cycle regulators, apoptosis, and oncogenic signaling pathways.[12,13] PIN1 interacts with the HBx protein and enhances its transcriptional activity.[14] Notably, PIN1-knockdown was shown to sensitize HCC cells to sorafenib treatment, underscoring its role in therapeutic resistance.[15] Furthermore, SENP1 was reported to enhance PIN1 activity via deSUMOylation in breast cancer,[16] suggesting a potential regulatory axis involving SENP1, PIN1, and the HBx protein in HCC that remains to be elucidated.

In this study, we demonstrate that HBx-induced SENP1 expression regulates levels of OCT4 and PIN1, promoting CSC-associated properties *in vitro* and *in vivo*. There properties include tumor sphere formation, the epithelial-mesenchymal transition (EMT), tumor growth, metastasis, and drug refractoriness. Our findings demonstrate that SENP1 is a key factor promoting cancer stemness in HBV-related HCC and may contribute to HCC recurrence and metastasis after surgery.

## Materials and methods

### Cell lines and HCC tissues

HepG2 (HBV-negative; BCRC, Taiwan), Huh7 (HBV-negative; JCRB, Japan), Hep3B (HBV-positive; BCRC), PLC5 (HBV-positive; BCRC), HepG2215 (derived from HepG2 by stable transfection with the HBV genome; kindly provided by Dr. Jun-Jen Liu, Institute of Medical Biotechnology, Taipei Medical University, Taipei, Taiwan), and Mahlavu (HBV-negative; kindly provided by Dr. Muh-Hwa Yang, Institute of Clinical Medicine, National Yang Ming Chiao Tung University, Taipei, Taiwan) HCC cell lines were used in this study. Sorafenib-resistant cell lines (HepG2215_R and Mahlavu_R) were established by gradually exposing parental cells (HepG2215 and Mahlavu) to increasing concentrations of sorafenib (Cell Signaling Technology, Boston, MA, USA). The resistant cell lines were subsequently maintained in medium containing 10 μM sorafenib.

Patients' frozen HCC tissues and corresponding formalin-fixed, paraffin-embedded HCC tissues were obtained from Chang Gung Memorial Hospital (Chiayi, Taiwan). This study was approved by the Institutional Review Board of Chang Gung Medical Foundation (IRB approval no. 101-5232B).

A detailed description of the materials and methods is provided in Supplementary Materials.

## Results

### SENP1 expression is associated with tumor recurrence, the pluripotent ESC signature, and poor prognoses in HCC

To evaluate the clinical relevance of SENP1 in HCC, *SENP1* mRNA levels were assessed in paired tumor (T) and adjacent peritumor (PT) tissues from 211 patients. Levels of *SENP1* mRNA were classified as high (T/PT ≥2) or low (T/PT < 2) (Table 1). Patients with high SENP1 levels exhibited a poorer OS (median survival: 69.2 *vs.* 172.8 months, $p$ <0.001) and shorter DFS (median survival: 15.8 *vs.* 39.7 months, $p$ = 0.004) (Fig. 1A), and high SENP1 expression was significantly associated with advanced TNM stages (Fig. 1B, Table 1). Elevated *SENP1* expression was further validated in the GSE76427 cohort,[17] showing significantly higher *SENP1* mRNA levels in tumor tissues *vs*. adjacent non-tumor tissues (Fig. 1C). Additionally, a significant and positive correlation was observed between *SENP1* expression and cancer stemness, which was also indicated by results of a gene set-enrichment analysis targeting HCC recurrence and embryonic stem cell (ESC) signatures (Fig. 1D). Consistently, *SENP1* mRNA levels were correlated with stemness/EMT markers (*OCT4*, *SNAIL*, *TWIST*) in tissues from 211 patients with HCC we further examined (Fig. 1E). These clinical observations highlight the potential role of SENP1 in HCC tumor recurrence and metastasis.

### Expression of SENP1 and CSC-associated OCT4, CD133, and EMT-related factors are highly correlated with early tumor recurrence in HBV-related HCC

We previously reported elevated OCT4 expression in tumors, especially in HBV-related HCC.[7] Given the link between SENP1 and stemness/EMT markers, we analyzed expression profiles of *SENP1*, *OCT4*, *SNAIL*, and *TWIST* across 211 HCC samples of various etiologies. As shown in Fig. 2A, patients with HBV-HCC (n = 83) exhibited significantly higher mRNA levels of these genes compared to NBNC-HCC (n = 23), HCV-HCC (n = 79), and BC-HCC (n = 26), indicating a strong correlation between *SENP1* and *OCT4* in HBV-HCC. To evaluate the prognostic significance, patients were stratified by gene expression and their DFS was analyzed within 24 months. A Kaplan-Meier analysis demonstrated that elevated mRNA levels of *SENP1* (7.8 *vs.* 15.7 months, $p$ = 0.003), *OCT4* (7.8 *vs.* 16.7 months, $p$ <0.001), *SNAIL* (8.6 *vs.* 15.7 months, $p$ = 0.012), and *TWIST* (8.2 *vs.* 15.5 months, $p$ = 0.028) were significantly associated with a shorter DFS (Fig. 2B). Immunohistochemical (IHC) staining confirmed significantly higher expression of SENP1 and the OCT4 and CD133 stemness-related proteins in HBV-HCC tumor tissues compared to NBNC-HCC tumor tissues (Fig. 2C, D). A positive correlation was observed between SENP1, OCT4, and CD133 protein levels (Fig. 2E).

### SENP1 regulates CSC-associated properties in HCC

CSC-associated properties have been well reported to involve expression of stemness-related markers such as OCT4 and CD133, and EMT-related N-cadherin, SNAIL, and TWIST, and with the ability to form tumor secondary spheres.[7,18] To investigate the effect of SENP1 on CSC-associated properties in HCC, overexpression and silencing approaches were respectively applied to HCC cell lines with low (Huh7 and PLC5) and high (HepG2 and Hep3B) endogenous SENP1 expression (Fig. S1). SENP1 overexpression in Huh7 and PLC5 cells significantly upregulated OCT4-and EMT-associated proteins such as N-cadherin, SNAIL, and TWIST (Fig. 3A), whereas the silencing of SENP1 expression in HepG2 and Hep3B cells reduced levels of these markers (Fig. 3B).

In the secondary sphere-formation assay, the effect of OCT4 was utilized as a positive control. As depicted in Fig. 3C, OCT4 overexpression in HepG2 cells led to increases in both the size and number of secondary tumor spheres, whereas suppression of OCT4 expression inhibited sphere formation. Similar to the effects of OCT4, SENP1 overexpression notably enhanced the formation of secondary tumor spheres, and

**Table 1. Variables associated with high and low SENP1 expression in HCC.**

| | SENP1 High[a] (n = 40) | SENP1 Low[a] (n = 171) | *p* value[b] |
|---|---|---|---|
| Gender | | | 0.940 |
| Male | 33 (73.3%) | 139 (72.8%) | |
| Female | 12 (26.7%) | 52 (27.2%) | |
| Age (mean ± SD) | 57.6 ± 13.2 | 61.9 ± 10.7 | 0.236[c] |
| HBV | **24 (60.0%)** | **59 (34.5%)** | **0.003*** |
| HCV | 10 (25.0%) | 69 (40.4%) | 0.071 |
| Bilirubin ≥1.2 mg/dl | 8 (20.0%) | 38 (22.2%) | 0.759 |
| Albumin <3.5 g/dl | 9 (22.5%) | 30 (17.5%) | 0.467 |
| ALT ≥35 U/L | 22 (55.5%) | 103 (60.23%) | 0.606 |
| PT, INR ≥1.2 | **5 (12.5%)** | **6 (3.5%)** | **0.021*** |
| AFP ≥400 ng/ml | 12 (30.0%) | 33 (19.3%) | 0.137 |
| ICG, retention rate ≥15% | 7 (17.5%) | 28 (16.4%) | 0.863 |
| TNM stage | | | **<0.001*** |
| Stage 1/2 | **28 (70.0%)** | **158 (92.4%)** | |
| Stage 3/4 | **12 (30.0%)** | **13 (7.6%)** | |
| Multiple tumors | 5 (12.5%) | 16 (9.4%) | 0.550 |
| Child-Pugh class | | | 0.193 |
| Class A | 40 (100%) | 164 (95.8%) | |
| Class B | 0 (0%) | 7 (4.2%) | |
| Complete tumor capsule | 9 (22.5%) | 47 (27.5%) | 0.520 |
| Microvascular invasion | **12 (30.0%)** | **22 (12.87%)** | **0.008*** |
| Macrovascular invasion | 2 (5.0%) | 4 (2.3%) | 0.362 |
| Cut margin free | 36 (90.0%) | 165 (96.5%) | 0.082 |
| Differentiation | | | 0.149 |
| Grade 1/2 | 11 (27.5%) | 68 (39.8%) | |
| Grade 3/4 | 29 (72.5%) | 103 (60.2%) | |
| Tumor size ≥3 cm | 27 (67.5%) | 101 (59.1%) | 0.326 |
| Satellite nodules | 10 (25.0%) | 24 (14.0%) | 0.090 |
| OCT4 expression (≥2x) | **38 (95.0%)** | **11 (6.4%)** | **<0.0001*** |
| SNAIL expression (≥2x) | **34 (85.0%)** | **12 (7.0%)** | **<0.0001*** |
| TWIST expression (≥2x) | **30 (75.0%)** | **12 (7.0%)** | **<0.0001*** |
| All three genes (≥2x) | **28 (70.0%)** | **2 (1.2%)** | **<0.0001*** |

*$p$ <0.05. n = 211. AFP, alpha-fetoprotein; ALT, alanine aminotransferase; ICG, indocyanine green; PT-INR, prothrombin time-international normalized ratio. Bold font indicates that the item is statistically significant.

[a]SENP1 High: SENP1 expression level of tumor/peritumor tissue ≥2; SENP1 Low: SENP1 expression level of tumor/peritumor tissue <2.

[b]Chi-squared test unless specified.

[c]Student's *t* test.

silencing of SENP1 expression mitigated the enhanced effect (Fig. 3D). Consistently, wound-closure assays revealed an enhanced migratory ability upon overexpression of SENP1 (Fig. 3E), supporting its involvement in the EMT and cell motility. These findings strongly support the role of SENP1 in CSC-associated properties and highlight the close relationship between SENP1 and OCT4/EMT in HCC. To investigate the correlation between SENP1 and the CSC-related factor, CD133, magnetic cell sorting was used to isolate $CD133^{high}$ and $CD133^{-/low}$ fractions from HepG2 and PLC5 cells. Compared to $CD133^{-/low}$ cells, $CD133^{high}$ cells exhibited high expression levels of SENP1 and CSC-related proteins, including OCT4, N-cadherin, SNAIL, and TWIST (Fig. 3F).

To investigate the impact of SENP1 on the $CD133^{+}$ cell population, knockdown of SENP1 expression was performed in HepG2 cells. SENP1 silencing significantly reduced the ratio of the $CD133^{+}$ cell population (Fig. 3G). To further explore the role of SENP1 in $CD133^{+}$ cell self-renewal, $CD133^{high}$ HepG2 cells were transduced with short hairpin (sh)SENP1 and cultured for 7 days. Results demonstrated that the silencing of SENP1 expression considerably decreased the $CD133^{+}$ cell population in HepG2 cells compared to the vector control group (Fig. 3H). Results indicated that SENP1 plays an important role in the self-renewal of $CD133^{+}$ HepG2 cells, thus maintaining the CSC population.

## HBx increases CSC-associated properties through regulation by SENP1 in HCC

The HBx protein was demonstrated to increase OCT4 expression and drive the pathogenesis of HBV-related HCC.[10] Given elevated SENP1/OCT4 expression in HBV-related HCC, we investigated the HBx protein's effect on SENP1 and its downstream impact on stemness features in HCC cells, including OCT4, EMT markers, secondary sphere formation, and migration/invasion.

HBx was overexpressed in HBV-negative (Huh7 and HepG2) and HBV-positive (Hep3B and PLC5) HCC cell lines. HBx significantly increased SENP1, OCT4, N-cadherin, SNAIL, and TWIST expression, while slightly decreasing E-cadherin expression (Figs. 4A, and S2A). Immunofluorescence staining results showed that cells with high levels of HBx-GFP expression had higher SENP1 expression compared to cells without HBx-GFP expression (Fig. 4B, SENP1 in red, and HBx-GFP in green, as indicated by arrowheads). Additionally, HBx overexpression induced a morphological transition in HepG2 cells from an epithelial-like to a mesenchymal-like phenotype, which was reversed upon SENP1 silencing (Fig. S2B).

To further investigate whether HBx regulates stemness and the EMT via SENP1, HepG2 cells were transduced with HBx-GFP or control-GFP vectors, with or without SENP1-knockdown

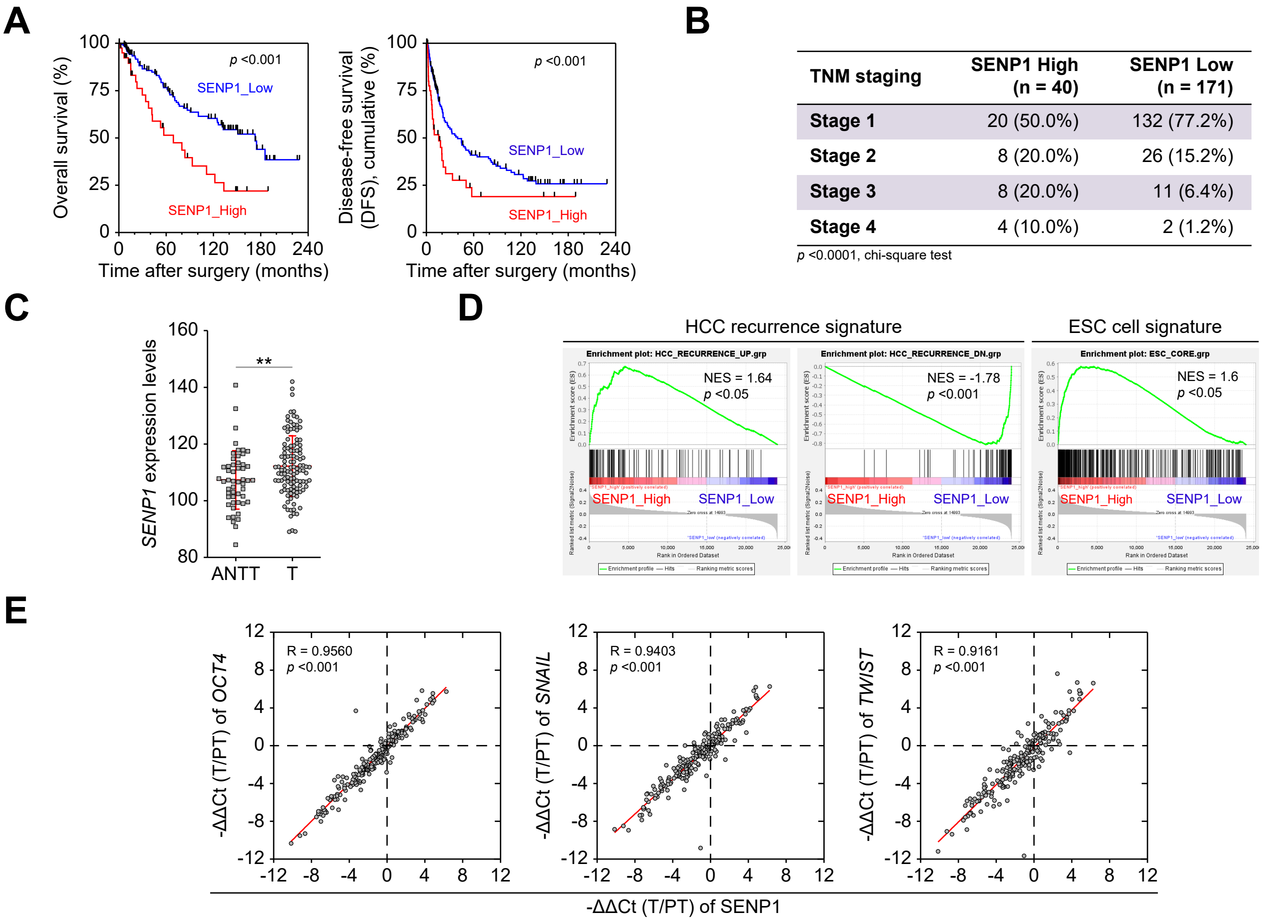


| TNM staging | SENP1 High (n = 40) | SENP1 Low (n = 171) |
|---|---|---|
| **Stage 1** | 20 (50.0%) | 132 (77.2%) |
| **Stage 2** | 8 (20.0%) | 26 (15.2%) |
| **Stage 3** | 8 (20.0%) | 11 (6.4%) |
| **Stage 4** | 4 (10.0%) | 2 (1.2%) |

**Fig. 1. *SENP1* expression is associated with tumor recurrence, a pluripotent embryonic stem cell signature, and poor prognosis in HCC.** (A) Kaplan-Meier curve for OS and DFS after HCC resection for assessment of the transcriptional expression of *SENP1* in HCC tissues (n = 211, 40 SENP1_High and 171 SENP1_Low). (B) Statistics on TNM stages of patients with HCC by *SENP1* expression levels (n = 211). $p$ <0.0001, Chi-squared test. (C) *SENP1* expression in ANTT *vs.* T tissues from the GSE76427 GEO dataset. ** $p$ <0.01, Mann-Whitney test. (D) GSEA showing the correlation of *SENP1* with HCC recurrence (HCC_RECURRENCE_UP and HCC_RECURRENCE_DN) and embryonic stem cell signature (ESC_CORE) gene sets in HCC from the GSE76427 GEO dataset. (E) qPCR analysis of *SENP1*, *OCT4*, *SNAIL*, and *TWIST* in T and PT tissues; correlations presented as (–ΔΔCt of T/PT) ($n$ = 211). *** $p$ <0.001, Spearman's test. ANTT, adjacent non-tumorous tissue; DFS, disease-free survival; ESC, embryonic stem cell; GEO, Gene Expression Omnibus; GSEA, gene set-enrichment analysis; HCC, hepatocellular carcinoma; NES, normalized enrichment score; OS, overall survival; PT, peritumor; T, tumor.

(shSENP1#1 or shSENP1#2). SENP1 silencing markedly reduced HBx-induced expression of OCT4-and EMT-associated markers, including N-cadherin, SNAIL, and TWIST (Fig. 4C). The impact of HBx on CSC-associated properties was further assessed by analyzing secondary tumor sphere formation and the $CD133^+$ cell population in HepG2 cells. HBx overexpression significantly enhanced sphere formation (Fig. 4D) and increased the $CD133^+$ cell population (Fig. 4E), whereas SENP1-knockdown markedly inhibited these HBx-induced effects. Moreover, HBx promoted EMT-associated phenotypes, including enhanced cell migration and invasion, as shown by wound-closure and transwell assays. These phenotypes were effectively suppressed by SENP1 silencing (Fig. 4F, G).

## The HBx protein regulates levels of cell cycle-associated PIN1/cyclin D1 through SENP1

PIN1 inhibition enhances sorafenib sensitivity and suppresses HCC growth,[15] while its overexpression is frequently observed in HBV-related HCC.[14] To examine the clinical relationship between PIN1 and SENP1, IHC staining was performed on tumor tissues from patients with NBNC and HBV-related HCC. As shown in Fig. 5A, SENP1 and PIN1 protein levels were elevated in HBV-related HCC, with a strong positive correlation observed between the two (Fig. 5B).

The role of HBx/SENP1 in regulating PIN1 expression was further investigated. Consistent with data described earlier in this text, we found that HBx protein overexpression significantly increased SENP1 protein levels (Figs. 4E and 5C), and importantly, it effectively induced expression of the PIN1 and cyclin D1 proteins (Fig. 5C). The effect of the HBx protein on PIN1 expression was confirmed by immunofluorescence staining targeting PIN1, which revealed the clear co-localization of HBx-GFP (indicated by GFP) and PIN1 (indicated by red fluorescence) in HCC cells. Higher HBx expression was correlated with more-intense PIN1 immunostaining. In Fig. 5D, an arrowhead points to an HCC cell showing low levels of both the HBx and PIN1 proteins.

Additionally, to investigate the upstream regulatory role of SENP1 in PIN1 protein expression, HA-SENP1 was

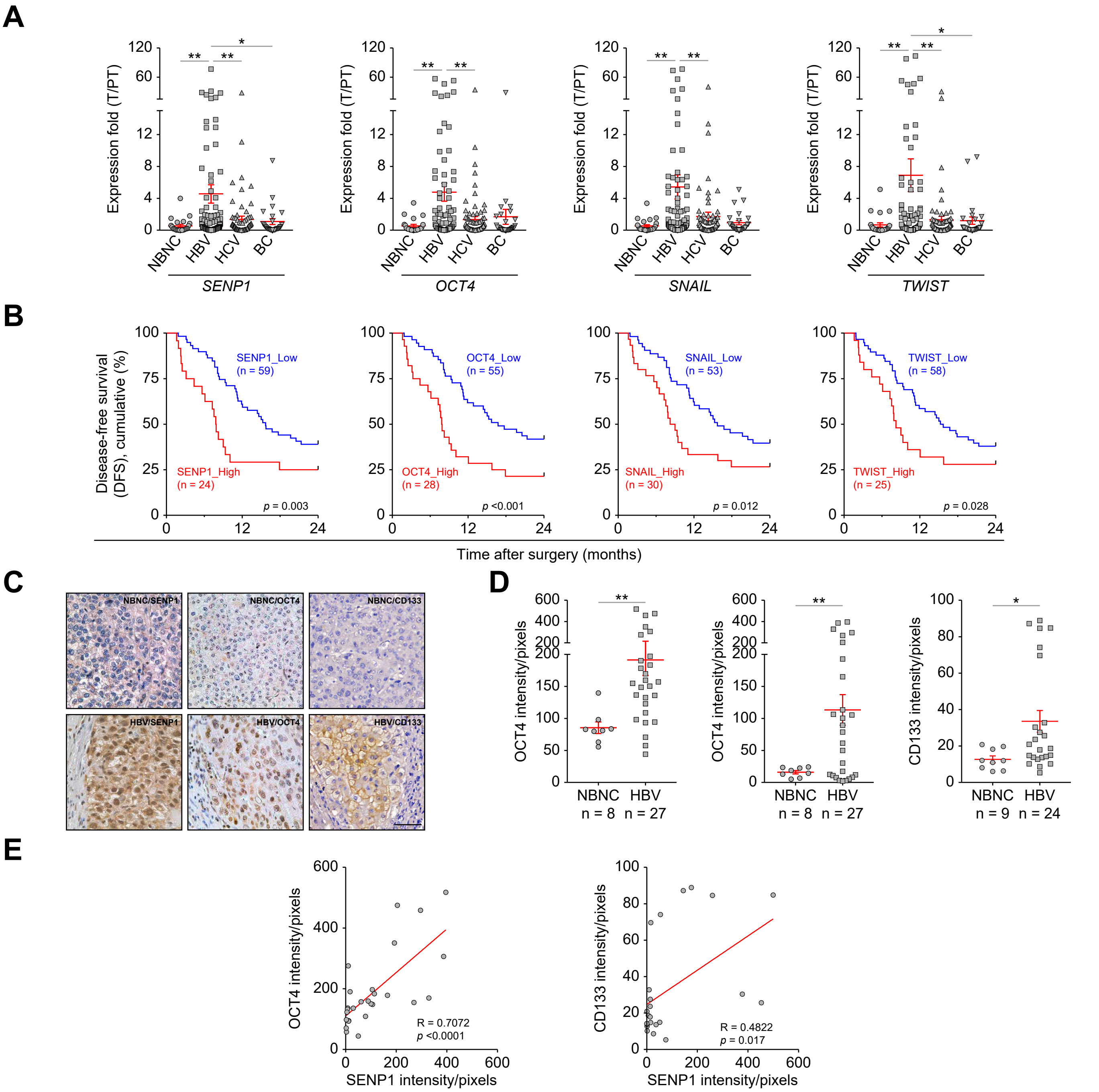


**Fig. 2. Positive correlations of *SENP1*, *OCT4*, *CD133*, *SNAIL*, and *TWIST* expression levels with early tumor recurrence in HBV-related HCC.** (A) qPCR analysis of *SENP1*, *OCT4*, *SNAIL*, and *TWIST* in paired T and PT tissues from patients with HCC of different etiologies: NBNC (n = 23), HBV (n = 83), HCV (n = 79), and BC (n = 26). *$p$ <0.05, ** $p$ <0.01, Mann-Whitney test. (B) Kaplan-Meier analysis of DFS (early tumor recurrence, 24 months) in HBV-related HCC (n = 83) based on transcriptional expression of the indicated genes. (C) Representative immunohistochemical staining images of SENP1, OCT4, and CD133 in tumor sections from patients with NBNC- and HBV-related HCC. Scale bars = 100 µm. (D) Quantitative data of (c) are shown as intensity/pixel. * $p$ <0.05, ** $p$ <0.01, Mann-Whitney test. (E) Correlations of protein levels (intensity/pixel) of SENP1 with OCT4 (n = 27) and CD133 (n = 24) in HBV-HCC tumor sections. Spearman's test. BC, HBV and HCV; DFS, disease-free survival; HCC, hepatocellular carcinoma; NBNC, non-HBV and non-HCV; PT, peritumor; T, tumor.

overexpressed in HepG2 cells to assess the induction of PIN1 and cyclin D1. As shown in Fig. 5E, SENP1 increased PIN1 and cyclin D1 expression in HepG2 cells. Furthermore, the role of SENP1 in HBx-PIN1/cyclin D1 regulation was investigated in HepG2 cells transfected with HBx-GFP alone or in combination with SENP1 shRNA. As illustrated in Fig. 5F, HBx-GFP overexpression significantly increased protein levels of SENP1, PIN1, and cyclin D1, and shSENP1, effectively attenuating the effect of HBx-GFP. These results strongly support the regulatory role of HBx/SENP1 in PIN1 expression in HCC cells.

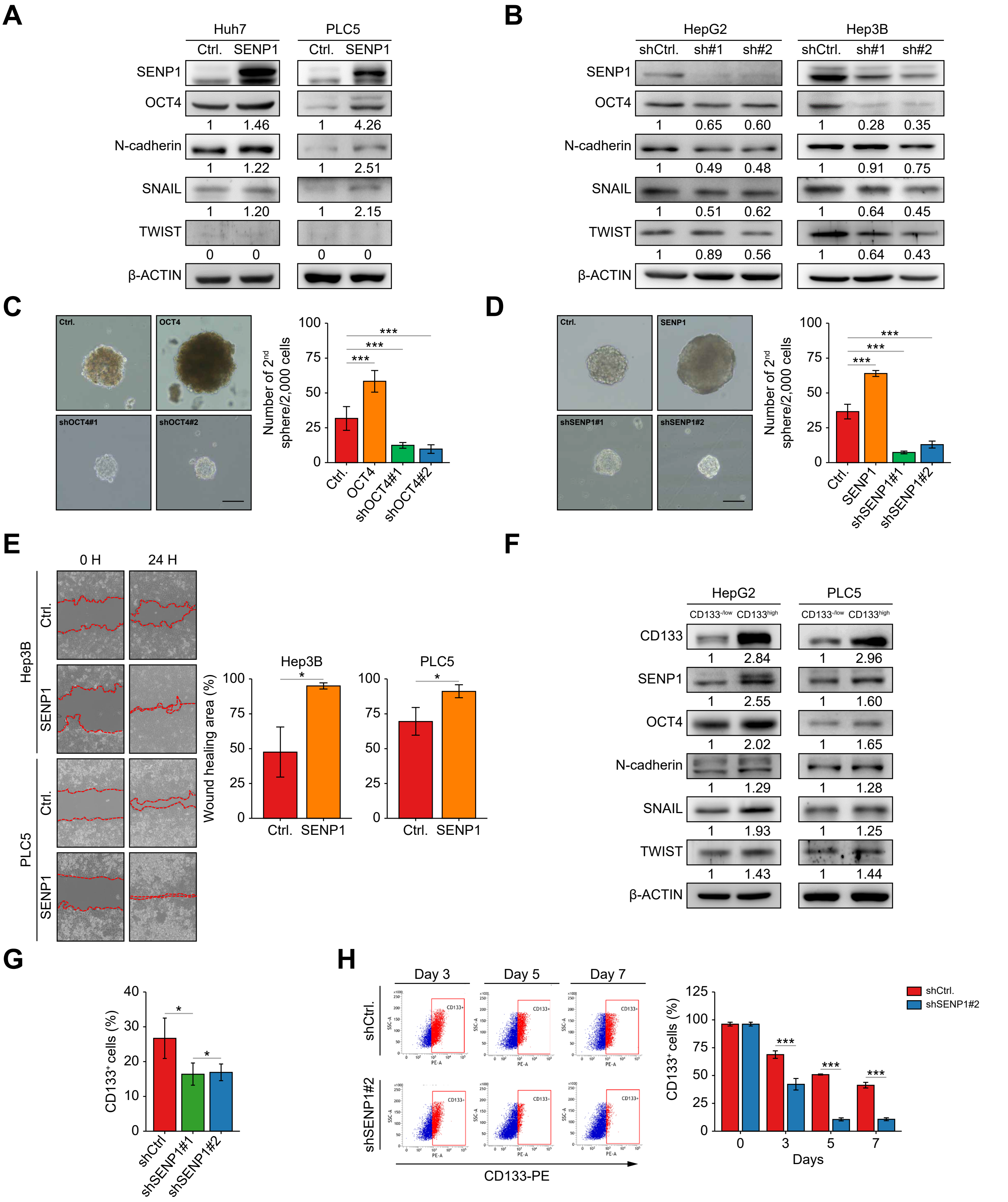


**Fig. 3. SENP1 regulates OCT4 expression, the EMT, tumor sphere formation, and the CD133$^+$ cell population in HCC.** (A,B) Western blot analysis of SENP1, OCT4, N-cadherin, SNAIL, and TWIST in Huh7 and PLC5 cells overexpressing SENP1 (A) and HepG2 and Hep3B cells with SENP1-knockdown (shSENP1#1 and shSENP1#2) (B). (C,D) Secondary tumor sphere-formation assay in HepG2 cells with OCT4 (C) or SENP1 (D) overexpression or silencing. Spheres of >100 μm were quantified (right panels). Scale bars = 100 μm. (E) Wound-closure assay to assess the effect of SENP1 overexpression on the migration of Hep3B and PLC5 cells at

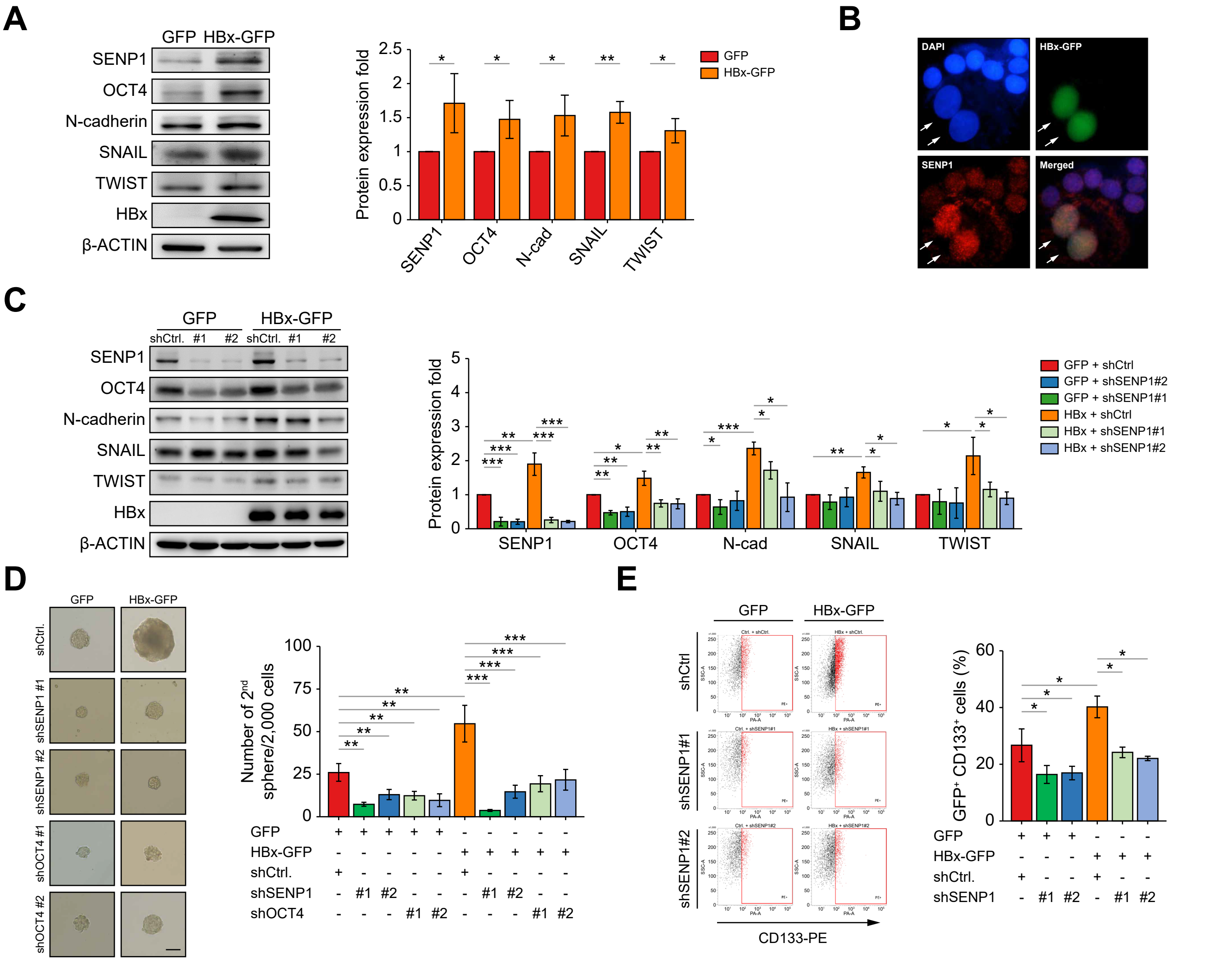


**Fig. 4. HBx increases CSC-associated properties through SENP1 regulation in HCC.** (A) Western blot analysis of indicated proteins in HepG2 cells transduced with GFP or HBx-GFP expression (a); quantified protein levels are shown in (b). (B) Co-localization of the HBx protein-GFP (green), SENP1 (red), and DAPI (blue) in HepG2 cells. An arrowhead indicates a cell with HBx-GFP expression. (C) Western blot of indicated proteins in GFP-HepG2 and HBx-HepG2 cells with or without SENP1-knockdown (shSENP1#1 and shSENP1#2) (a); quantified protein levels are shown in (b). (D) Secondary tumor sphere formation assay in GFP-HepG2 and HBx-HepG2 cells with or without silencing SENP1 or OCT4 expression (a); quantification of spheres >100 µm is shown in (b). (E) Flow cytometric analysis of $GFP^+CD133^+$ populations in HepG2 cells (a); quantified in (b). (F) Wound-closure assay to assess the effect of SENP1 silencing on HBx-induced cell migration of HepG2 cells at 0, 12, and 24 h (a); quantified in (b). (G) Matrigel-coated transwell assay evaluating cell invasion under indicated conditions (a); quantified in (b). * $p$ <0.05, ** $p$ <0.01, *** $p$ <0.001, Student's $t$ test. Scale bars = 100 µm. Western blot analyses were conducted across all conditions, with β-ACTIN as the loading control. CSC, cancer stem cell; HBx, HBV X protein; HCC, hepatocellular carcinoma; shSENP1, small hairpin RNA targeting SENP1.

## HBx-SENP1 promotes tumor growth and metastasis in an orthotopic HCC xenograft model

To validate the *in vitro* findings of HBx/SENP1-mediated regulation of OCT4 and EMT-related properties (Table 1, Figs. 1-4), an orthotopic liver xenograft model was established using HepG2 cells stably expressing the GFP or HBx protein, with or without SENP1 shRNA (shSENP1). Control shRNA (shCtrl) was used as the control group. HCC tumor-bearing mice were divided into four groups: GFP-shCtrl (control group, n = 5), GFP-shSENP1 (control group with shSENP1, n = 6), HBx-shCtrl (HBx group, n = 8), and HBx-shSENP1 (HBx group with shSENP1, n = 12). Notably, mice in the GFP-shCtrl and HBx-shCtrl groups exhibited significant reductions in body weight compared to those in the SENP1-silenced groups (GFP-shSENP1 and HBx-shSENP1) (Fig. 6A).

0 and 24 h. Quantified data are shown (right panel). (F) Western blot of CD133, SENP1, OCT4, N-cadherin, SNAIL, and TWIST protein levels in sorted $CD133^{high}$ and $CD133^{-/low}$ HepG2 and PLC5 cells. (G) Flow cytometric analysis of $CD133^+$ cell populations in HepG2 cells. (H) The $CD133^+$ cell population was analyzed by flow cytometry in sorted $CD133^{high}$ cells from HepG2 cells after being cultured for the indicated time. Quantification is shown (right panels). β-ACTIN served as the loading control; quantified values are shown below. * $p$ <0.05, *** $p$ <0.001, Student's $t$ test. Ctrl, control vector; EMT, epithelial-mesenchymal transition; HCC, hepatocellular carcinoma; shCtrl, shRNA of LacZ gene; shSENP1, small hairpin RNA targeting SENP1.

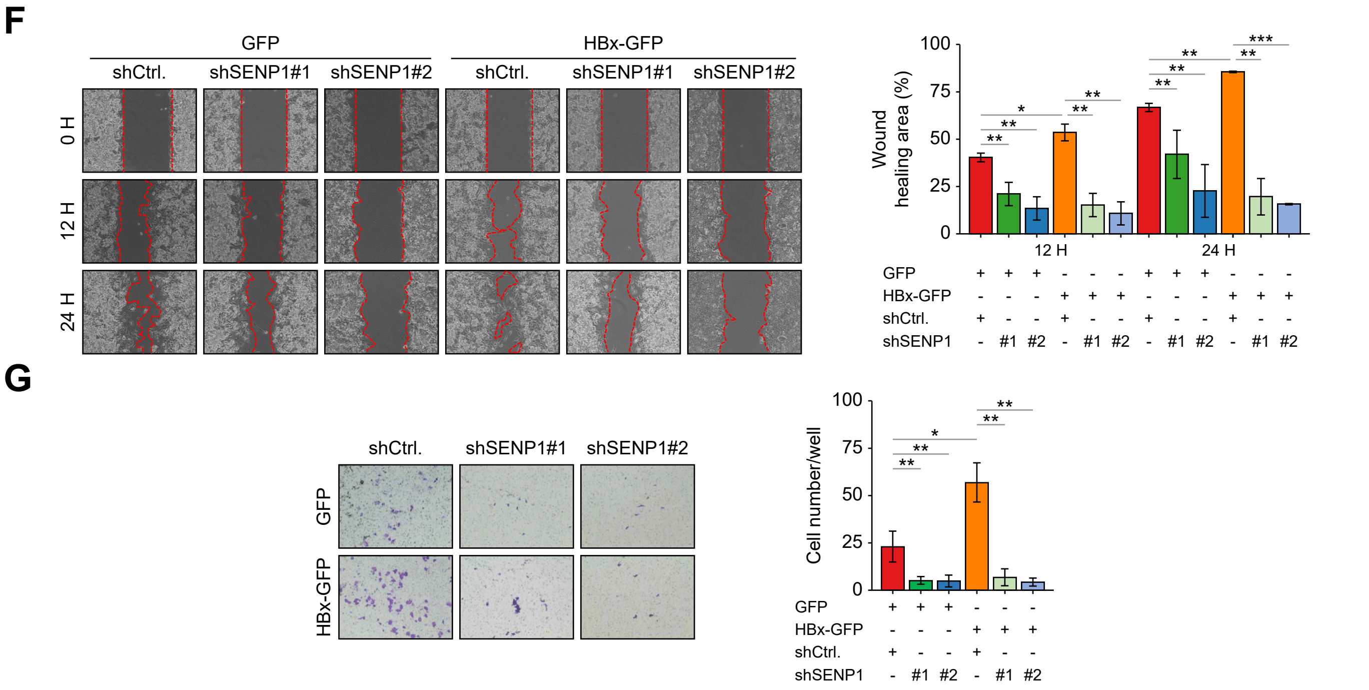


**Fig. 4. (continued).**

Tumor sizes in each group were monitored by bioluminescence intensity. As shown in Fig. 6B, bioluminescence imaging demonstrated a progressive increase in signal intensity in tumors in both the GFP-shCtrl and HBx-shCtrl groups, with the HBx-shCtrl group exhibiting a markedly stronger signal. In contrast, SENP1 silencing effectively suppressed tumor progression, as evidenced by the bioluminescence intensity of liver tissues in the GFP-shSENP1 and HBx-shSENP1 groups at 8 weeks post-implantation of HCC cells (Fig. 6B). Quantitative data are shown in Fig. 6C. Further gross examination of liver tissues at 8 weeks post-implantation revealed discernible HCC tumors in the experimental group without SENP1 silencing (Figs. 6D and S3, GFP-shCtrl and HBx-shCtrl groups). Tumor sizes and liver masses were notably greater in the HBx-shCtrl group compared to the GFP-shCtrl group, indicating enhanced tumorigenicity (Fig. 6D). Consistent with the bioluminescence results in Fig. 6B, SENP1-silenced groups (GFP-shSENP1 and HBx-shSENP1) exhibited either markedly smaller liver tumors or no visible tumor formation, demonstrating that SENP1 silencing substantially impaired tumor outgrowth *in vivo* (Figs. 6D and S3). These observations were further verified by H&E staining of representative histological sections corresponding to HCC tumors in Fig. 6D (Fig. 6E). Additionally, H&E staining revealed lung metastases in tumor-bearing mice (Fig. 6F, Table S3). The HBx-shCtrl group showed more-extensive pulmonary lesions compared to the other groups, based on a quantitative analysis of the lung metastatic area (Fig. 6G). Importantly, liver tumors exhibited invasion into the fibrous capsule in mice with lung metastasis (Fig. 6H). In contrast, no such invasion was observed in the SENP1-silenced group (Fig. 6H). Immunostaining results revealed elevated levels of SENP1, OCT4, CD133, N-cadherin, and PIN1 proteins in the HBx-shCtrl group, whereas these proteins were markedly reduced in both the liver and lung metastatic tumors in the SENP1-silenced groups (Fig. 6H, I).

### HBx-SENP1 contributes to sorafenib resistance in vitro and in vivo

Sorafenib refractoriness remains a key therapeutic concern in HCC. To evaluate whether SENP1 modulates sorafenib responsiveness, we treated Hep3B, PLC5, HepG2215, and sorafenib-resistant HepG2215_R and Mahlavu_R cells with increasing concentrations of sorafenib in the presence of various doses of a SENP1 inhibitor (SENP1-IN-3). The individual $IC_{50}$ (50% inhibitory concentration) values for sorafenib and the SENP1 inhibitor in each HCC cell line are presented in Fig. S4. Dose-response profiles for combined treatments with sorafenib and the SENP1 inhibitor are plotted in Fig. 7A, and corresponding $IC_{50}$ values were calculated and are summarized in Fig. 7B. These results demonstrate that SENP1 inhibition sensitized both naïve and sorafenib-resistant HCC cells to sorafenib treatment. To further assess the impact of SENP1 on the sorafenib response, HCC xenograft-bearing mice were treated with sorafenib and monitored by bioluminescence imaging. As shown in Fig. 7C,D, the GFP-shCtrl group (n = 4) exhibited a significant reduction in the tumor signal after 1 week of treatment, whereas the HBx-shCtrl group (n = 7) showed a marked increase in the tumor burden over the same period. After 4 weeks of sorafenib treatment, no therapeutic response was observed in the HBx-shCtrl group, as evidenced by continued tumor progression. In contrast, two mice in the GFP-shCtrl group demonstrated sustained suppression of tumor growth throughout the treatment period (Fig. 7C,D). Importantly, no apparent tumor progression was observed in

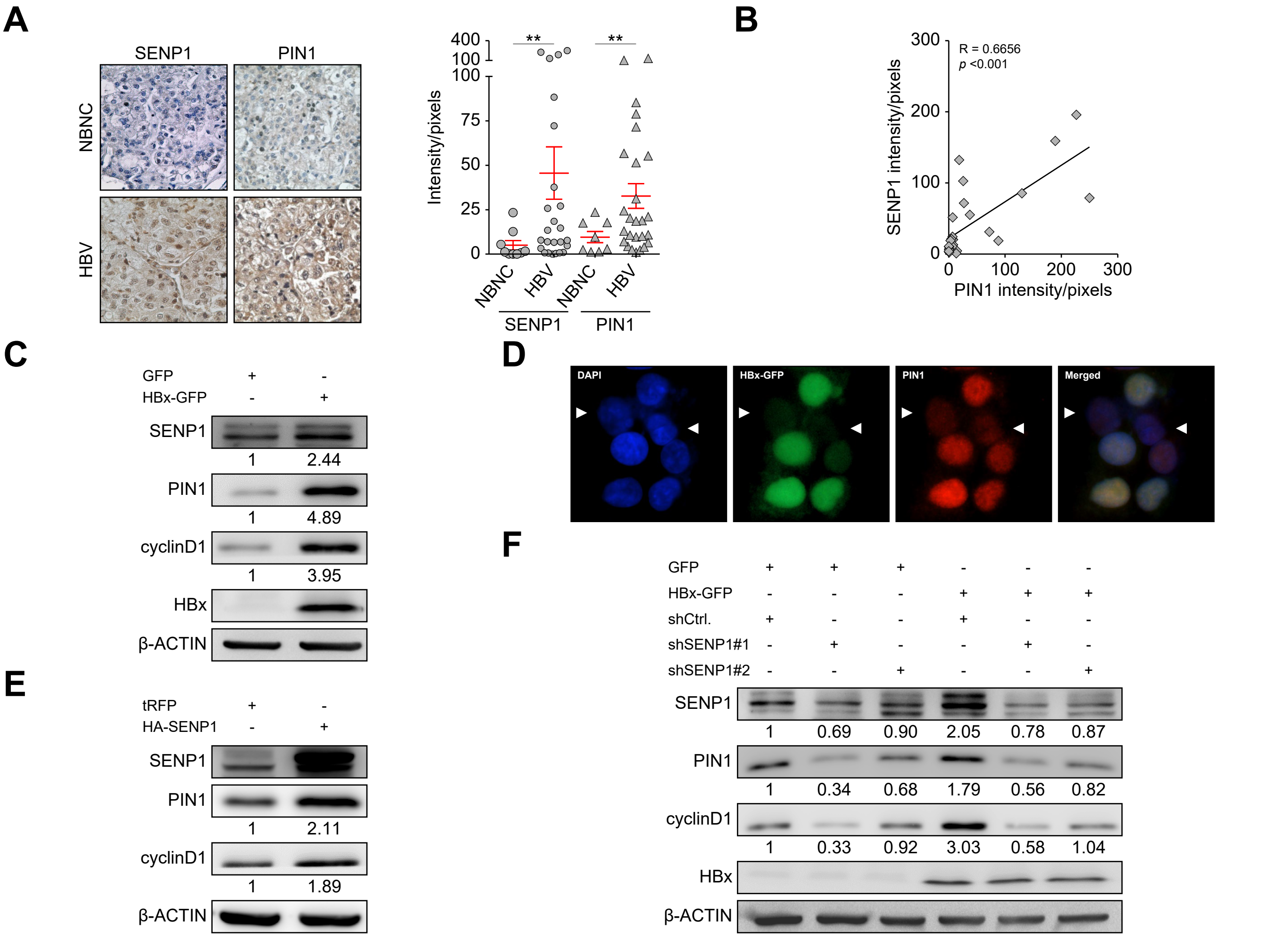


**Fig. 5. HBx increases PIN1 and cyclin D1 expressions through SENP1 in HBV-related HCC.** (A) Immunohistochemical staining of SENP1 and PIN1 in representative tumor sections from patients with NBNC-HCC (n = 8) and HBV-HCC (n = 25). Quantification of protein levels (intensity/pixel) is shown on the right. Scale bars = 100 μm ** *p* <0.01, Mann-Whitney test. (B) Correlation of protein levels of SENP1 and PIN1 in HBV-HCC tumor sections (n = 25). *p* <0.001, Spearman's test. (C) Western blot analysis of SENP1, PIN1, cyclin D1, and HBx in HepG2 cells overexpressing HBx-GFP. (D) Co-localization of the HBx protein-GFP (green), PIN1 (red), and DAPI (blue) in HepG2 cells. The arrowhead indicates a non-transduced cell. (E) Western blot analysis of SENP1, PIN1, and cyclin D1 in SENP1-overexpressing HepG2 cells. (F) Western blot analysis of the indicated proteins in GFP- and HBx-GFP HepG2 cells with or without SENP1-knockdown (shSENP1#1 and shSENP1#2). β-ACTIN served as the loading control; quantified values are shown below. HBx, HBV X protein; HCC, hepatocellular carcinoma; NBNC, non-B non-C; shSENP1, small hairpin RNA targeting SENP1.

the SENP1-silenced groups throughout the treatment period, indicating effective suppression of tumor growth (Fig. 7C,D).

## Discussion

OCT4 is widely expressed in various tumor types and strongly linked to CSC-related properties.[19] A meta-analysis also revealed that OCT4 expression was correlated with tumor sizes, tumor numbers, cell differentiation, and the TNM stage, and that OCT4 expression was associated with poor 3- and 5-year OS and DFS rates in HCC.[20] However, the molecular mechanism through which OCT4, which is closely related to the pluripotency of ESCs, is re-expressed in somatic cancer cells remains unknown. In this study, we demonstrated that the HBx protein promotes CSC-associated properties, such as OCT4 upregulation, through regulation by SENP1. We also found that SENP1 and OCT4/EMT expression levels were positively correlated with early tumor recurrence in patients with HCC. The co-expression of HBx and SENP1 may contribute to the upregulation of OCT4/EMT expression in HCC. SENP1-knockdown effectively suppressed HBx-induced OCT4/EMT expression and inhibited intrahepatic and pulmonary metastases *in vivo*, suggesting that SENP1 could serve as a prognostic marker and a potential therapeutic target in HBV-related HCC.

Niche and epigenetic regulation are regarded as pivotal factors in OCT4 functionality.[19] DNA methylation at CpG sites in promoter and exonic regions modulates OCT4 transcription in trophoblast cells and ESCs.[21,22] In somatic cancers such as glioblastomas, aberrant regulation by DNA methyltransferase (DNMT) can trigger CSC development through OCT4 reprogramming.[23] Our previous studies demonstrated that patients with HBV-HCC have higher serum IL-6 concentrations, where IL-6 promotes CSC-associated properties by activating the IGF-

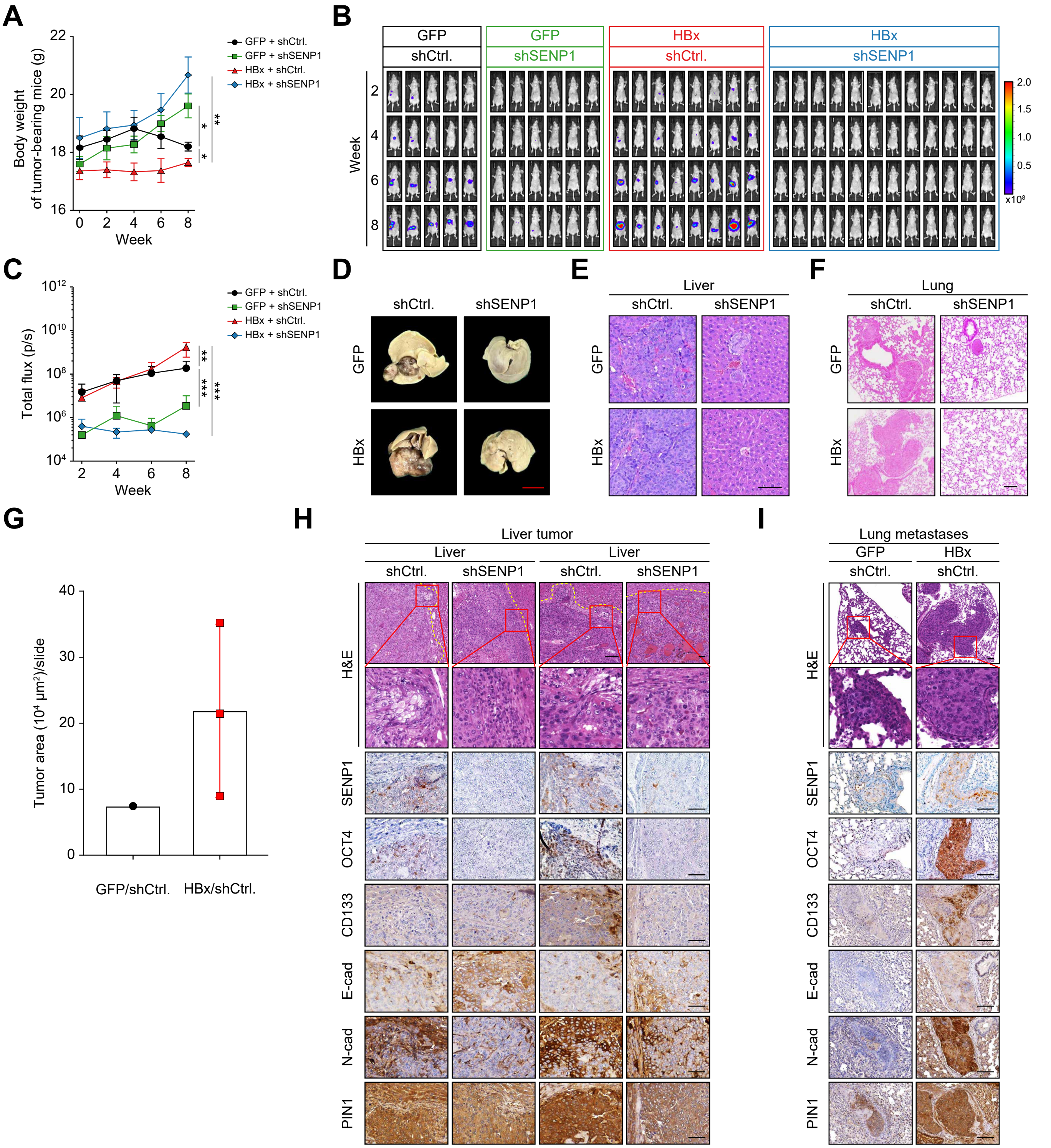


**Fig. 6. HBx-induced SENP1 expression promotes tumor growth, metastasis, and sorafenib refractoriness in an orthotopic HCC xenograft model.** (A) Body weights of mice in each group were monitored throughout the experiment: GFP-shCtrl (n = 5), GFP-shSENP1 (n = 6), HBx-shCtrl (n = 8), and HBx-shSENP1 (n = 12). Data from week 8 were analyzed using the Mann-Whitney *U* test. **p* <0.05, ***p* <0.01. (B) Representative bioluminescence images of orthotopic HCC xenografts from the GFP-shCtrl (n = 5), GFP-shSENP1 (n = 6), HBx-shCtrl (n = 8), and HBx-shSENP1 (n = 12) groups. Data from week 8 were analyzed using the Mann-Whitney *U* test. ***p* <0.01, ****p* <0.001. (C) Bioluminescence quantification of the tumor burden, expressed as total flux (photons/s). (D) Liver morphology of mice of the GFP-shCtrl, GFP-shSENP1, HBx-shCtrl, and HBx-shSENP1 groups at week 8 post orthotopic HCC cell injection. (E) Representative H&E staining of liver tumor xenograft tissues. (F) Representative lung images highlighting metastatic lesions in the GFP-shCtrl and HBx-shCtrl groups. (G) Quantification of metastatic tumor areas in lung tissue sections ($10^4$ μm$^2$/mouse/slide), corresponding to panel F. (H,I) Histological evaluation of liver tumors (H) and lung metastases (I) in the xenograft mouse model. H&E staining of representative liver tumor (H) and lung metastasis (I) tissue sections. The yellow dotted line in panel H highlights the fibrous capsule at the tumor margin;

1/IGF-1R signaling pathway.[7] IL-6/IGF-1R signaling-mediated OCT4 expression and sorafenib resistance may involve DNMT3b in HBV$^+$ HCC cells.[24] In this study, we found that the IL-6/STAT3 signaling pathway not only mediated OCT4 expression but also partially increased SENP1 expression in HCC cells with HBx expression (Fig. S5). Our previous data demonstrated that SENP1 stabilizes OCT4 protein levels,[11] and that OCT4 expression is modulated by IL-6/IGF-1R signaling.[7] Herein, we further validated that HBx-induced SENP1 contributes to IGF-1R upregulation in HepG2 cells (Fig. S6). Collectively, these findings indicate that IL-6 enhances OCT4 expression and CSC-associated properties in HBV-related HCC via a complex regulatory network involving IGF-1/IGF-1R, DNMT3b, and SENP1.

The HBx protein was shown to regulate epigenetic mechanisms, including micro (mi)RNA, DNA methylation, and histone modifications, which contribute to CSC features.[25] Previous studies indicated that miR-145 is downregulated in HBV-related HCC, which is potentially influenced by HBx.[26] MiR-145 was reported to target the 3′-untranslated region (UTR) of *OCT4* and *SENP1* mRNAs,[27,28] thereby suppressing their expression. Interestingly, OCT4 also binds to the miR-145 promoter and represses its transcription in human ESCs,[27] suggesting a potential positive feedback loop that may contribute to an enhancement of CSC properties. In this study, using our HBx-HepG2 cell model, we observed that HBx partially enhanced *SENP1* promoter activity and significantly suppressed miR-145-5p expression (Fig. S7A and B). Sequence alignment identified a putative miR-145-5p-binding site within the SENP1 3′-UTR (Fig. S7C), and luciferase assays confirmed that miR-145-5p overexpression significantly reduced SENP1 3′-UTR activity (Fig. S7D). Collectively, these findings demonstrated that HBx increases SENP1 expression by partially enhancing *SENP1* promoter activity and suppressing miR-145-5p expression, which counteracts its inhibitory effect on *SENP1* mRNA (Fig. S7E).

HBx influences several critical signaling pathways relevant to CSC-associated properties, including the Wnt/β-catenin, TGF-β1, NF-κB, Notch, Hedgehog, STAT3, and PI3K/AKT signaling pathways.[29] A study demonstrated that HBx upregulates CSC-related gene expression, such as *CD133* and *OCT4*, in OV6$^+$ CSCs in HCC through the β-catenin signaling pathway.[30] Another intriguing study found that HBx increases levels of OCT4 and MYC, facilitating the cellular reprogramming of induced pluripotent stem cells and potentially contributing to the formation of liver CSCs.[31] A recent study employed the CRISPR/Cas9 system to target the *HBx* gene; HBx-knockdown effectively reduced signatures of EMT and CSC genes in HBV-HCC cells.[32] In this study, in addition to demonstrating that SENP1 regulates OCT4 to enhance CSC properties, we also found that SENP1 modulated the self-renewal capacity of CD133$^{high}$ HepG2 cells *in vitro* (Fig. 3). Furthermore, *in vivo* animal experiments revealed that silencing SENP1 reduced HBx-enhanced CD133 expression (Fig. 6). To analyze the correlation between *CD133* mRNA (*PROM1*) and *SENP1* mRNA in HCC, we utilized the GSE76427 public dataset. As shown in Fig. S8, a significant positive correlation was observed between *PRMO1* and *SENP1* mRNA expression (n = 115). To further investigate, we examined protein levels of CD133 and SENP1 through IHC staining in HCC tissues, comprising eight NBNC-HCC and 24 HBV-HCC samples. Expression levels of SENP1 and CD133 were positively correlated in patients with HBV-HCC (Fig. 2C-E). Overall, our results further clarify the relationship between HBx and OCT4/CSC properties through regulation by SENP1.

Upregulation of SENP1 was recently reported in various cancer types, including breast cancer, lung cancer, prostate cancer, HCC, and colorectal cancer.[33] SENP1 overexpression disrupts the balance of SUMOylation by targeting specific proteins, significantly contributing to tumor progression and poor prognoses. In this study, we demonstrated for the first time that SENP1 is overexpressed in HBV-related HCC and is correlated with poor OS, poor DFS and extrahepatic metastasis, a critical feature of malignant tumors that considerably affects patient prognosis.

The EMT is a potential mechanism of tumor cell metastasis, and it recently emerged as an important regulator of CSC-associated properties in HCC.[34] Numerous studies identified the significant role of SENP1 in the EMT across various tumors. In HCC, SENP1 was demonstrated to promote hypoxia-induced cancer stemness by deSUMOylating HIF-1α, thereby establishing a positive feedback loop.[35] Additionally, SENP1 has been shown to regulate the hepatocyte growth factor-induced invasion and migration of HCC cells.[36] In our *in vitro* experiments, we demonstrated that SENP1 was associated with an increased EMT phenotype in HCC cells. Furthermore, *in vivo* experiments using an orthotopic xenograft model revealed a significant reduction in tumor metastasis following SENP1-knockdown. In addition, OCT4 is also involved in regulating the EMT. Our previous studies demonstrated that OCT4 increases levels of EMT-related factors, including SNAIL, TWIST, and SLUG, in HCC and human endometriosis.[37,38] These findings suggest that HBx-induced SENP1 and OCT4 may be a promising target for inhibiting the EMT and tumor metastasis in HBV-related HCC.

PIN1 is overexpressed in HCC, particularly in HBV-related HCC, and is associated with adverse features such as increased tumor sizes, intrahepatic metastasis, and poor prognoses.[12,14,39] Functionally, PIN1 interacts with cyclin D1 and β-catenin to modulate oncogenic pathways such as PI3K/Akt/mTOR and the EMT,[40] and was shown to stabilize the HBx protein and enhance its transactivation potential in HCC.[14] Recent evidence implicates PIN1 in therapeutic resistance, with its knockdown enhancing sorafenib sensitivity in HCC models.[15] SENP1-mediated deSUMOylation enhances PIN1 activity in breast cancer.[16] Consistent with previous findings, our study demonstrated increased expression and a significant correlation between SENP1 and PIN1 in HBV-related HCC. Notably, HBx promotes expression of PIN1 and cyclin D1 through SENP1, suggesting that SENP1 facilitates HBx/PIN1-driven transcriptional activity. Collectively, these findings provide compelling evidence that HBx may regulate PIN1 expression and HBx/PIN1 transactivation through SENP1-

the red boxed region is shown at higher magnification below. Immunohistochemical staining of SENP1, OCT4, CD133, E-cadherin, N-cadherin, and PIN1 was performed on representative samples from liver tumors and lung metastases. Scale bars = 50 μm. HBx, HBV X protein; HCC, hepatocellular carcinoma; shCtrl, control small hairpin RNA; shSENP1, small hairpin RNA targeting SENP1.

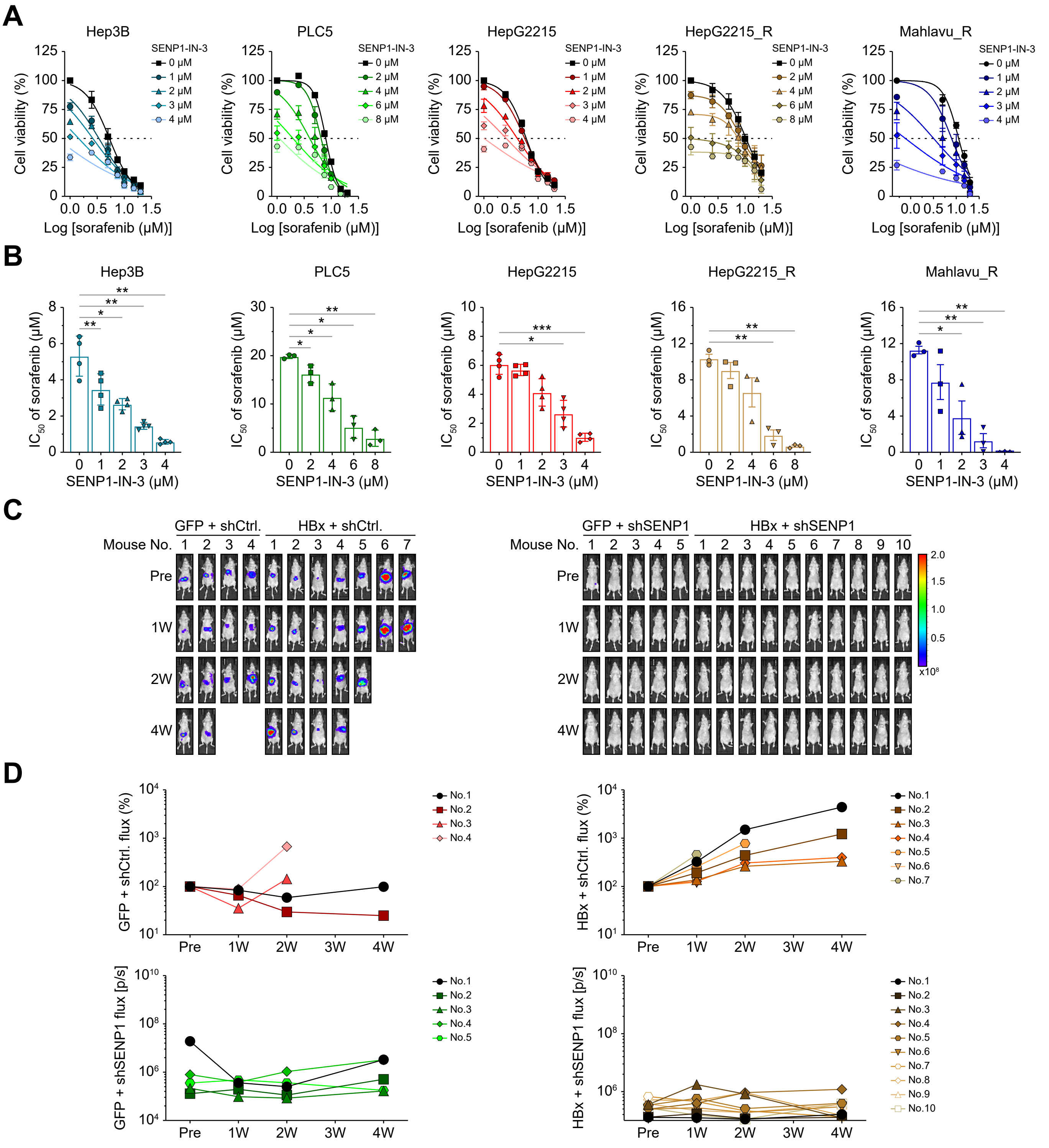

**Fig. 7. HBx/SENP1 signaling decreases cellular responsiveness to sorafenib *in vitro* and reduces tumor sensitivity in an *in vivo* xenograft model.** (A) Dose-response curves of sorafenib in Hep3B, PLC5, HepG2215, HepG2215_R, and Mahlavu_R HCC cell lines treated with increasing concentrations of SENP1-IN-3. Combination treatment was administered for 48 h, and cell viability was measured to assess the effect of SENP1-IN-3 on sorafenib sensitivity. (B) $IC_{50}$ values of sorafenib in five HCC cell lines following combination treatment with SENP1-IN-3, as calculated from dose-response curves in panel A. * $p$ <0.05, ** $p$ <0.01, *** $p$ <0.001, Student's $t$ test. (C) *In vivo* assessment of sorafenib response in four xenograft mouse groups: GFP-shCtrl (n = 4), GFP-shSENP1 (n = 5), HBx-shCtrl (n = 7), and HBx-shSENP1 (n = 10). Mice received intraperitoneal sorafenib treatment (30 mg/kg body weight, twice weekly) beginning at week 8 post-cell injection (pre-treatment). (D) The tumor burden was monitored weekly by bioluminescence imaging. Flux (photons/s) at week 8 was set to 100% as the baseline for normalization. The panel shows the tumor burden in individual mice. HCC, hepatocellular carcinoma; shCtrl, shRNA of LacZ gene; shSENP1, small hairpin RNA targeting SENP1.

mediated deSUMOylation in HBV-related HCC; thus, HBx promotes tumorigenesis.

Drug refractoriness is a major issue impeding the successful chemotherapeutic treatment of HCC. Many factors contribute to the development of drug resistance in HCC; for example, a hypoxic tumor microenvironment is known to play a pivotal role in promoting refractoriness to therapy.[41] Sorafenib, a first-line multikinase inhibitor approved for advanced HCC, was shown to modestly prolong median survival by 3–5 months.[42–44] SENP1 was implicated in cancer progression and may contribute to sorafenib resistance via its regulation of oncogenic proteins such as PIN1. A study revealed that PIN1-knockdown effectively increased sensitivity to sorafenib in HCC.[15] Our findings demonstrated that HBx enhances PIN1 and cyclin D1 expression through SENP1, supporting its role in HBx-driven drug refractoriness. To assess the therapeutic relevance, we evaluated combination treatment using sorafenib and a SENP1 inhibitor (SENP1-IN-3) across five HCC cell lines, including resistant models. This approach significantly reduced the effective dose of sorafenib (Fig. 7A,B). SENP1-knockdown restored sorafenib sensitivity in HBx-expressing tumors *in vivo* (Fig. 7C), indicating that SENP1 is a key modulator of drug responsiveness in HBV-related HCC.

In this study, we identified significant clinical associations of the SENP1-associated CSC- and EMT-related factors with poor OS and poor DFS in HBV-related HCC. We showed that HBV upregulates SENP1 expression through HBx, possibly by suppressing miR145 levels. This upregulation subsequently leads to increased expressions of CSC-associated factors (OCT4, CD133, and IGF-1R), EMT-associated factors (SNAIL and TWIST), and cell proliferation factors (PIN1 and cyclin D1). This promotes the self-renewal, migration, invasion, and sorafenib resistance of CSCs in HBV-related HCC. These findings highlight the crucial role of SENP1 in the progression of HBV-related HCC and suggest that SENP1 could serve as a prognostic marker and therapeutic target in this context.


## Affiliations

*[1]School of Respiratory Therapy, College of Medicine, Taipei Medical University, Taipei, Taiwan; [2]International PhD Program in Cell Therapy and Regenerative Medicine, College of Medicine, Taipei Medical University, Taipei, Taiwan; [3]Chen Wei-Tien Research Center of Thoracic Medicine, Taipei Medical University, Taipei, Taiwan; [4]Department of Biochemistry and Molecular Cell Biology, School of Medicine, College of Medicine, Taipei Medical University, Taipei, Taiwan; [5]Graduate Institute of Medical Sciences, College of Medicine, Taipei Medical University, Taipei, Taiwan; [6]TMU Research Center for Cell Therapy and Regeneration Medicine, Taipei Medical University, Taipei, Taiwan; [7]Core Laboratory of Good Tissue Practice, Office of Research and Development, Taipei Medical University, Taipei, Taiwan; [8]Department of Pathology, Chang Gung Memorial Hospital, Chiayi, Taiwan; [9]Graduate Institute of Microbiology, College of Medicine, National Taiwan University, Taipei, Taiwan; [10]Department of General Surgery, Chang Gung Memorial Hospital, Chiayi, Taiwan; [11]Division of Gastroenterology and Hepatology, Department of Internal Medicine, Chang Gung Memorial Hospital, Chiayi, Taiwan; [12]College of Medicine, Chang Gung University, Taoyuan, Taiwan; [13]School of Medicine, College of Medicine, National Sun Yat-sen University, Kaohsiung, Taiwan; [14]Center for Reproductive Medicine, Taipei Medical University Hospital, Taipei Medical University, Taipei, Taiwan*


## Abbreviations

BC, HBV and HCV; CSC, cancer stem cell; DFS, disease-free survival; EMT, epithelial-mesenchymal transition; ESC, embryonic stem cell; HBx, HBV X protein; HCC, hepatocellular carcinoma; NBNC, non-HBV and non-HCV; OS, overall survival; PIN1, peptidylprolyl isomerase 1; SENP1, SUMO-specific peptidase 1; SNAIL, snail family transcriptional repressor 1; TWIST, twist family bHLH transcription factor 1.


## Financial support

This work was supported by research grants from the National Science and Technology Council, Taiwan (grant nos.: MOST 106-2314-B-038-057 and MOST 107-2314-B-038-087-MY3 [to YC Wu]; NSC 102-2628-B-038-008-MY3, NSTC 113-2314-B-038-136 and NSTC 114-2314-B-038-012 [to YH Huang]; MOST 104-2314-B-182-073-MY2 and MOST 105-2628-B-182-013-MY3 [to TS Chang]), and by grants from the Chen Wei-Tien Research Found for Thoracic Medicine, Taipei Medical University (TMU 111-5431-001-400 [to YC Wu]).


## Conflict of interest

The authors have no conflicts of interest to declare.

Please refer to the accompanying ICMJE disclosure forms for further details.

## Authors' contributions

YH Huang and TS Chang initiated and supervised the research; YC Wu and YH Huang conceived and designed the project; YC Wu, YC Huang, YC Kuo, MHT Ngo, and YT Sung performed the *in vitro* experiments and data analysis; KF Lee and LM Kuo handled pathological tissue processing and tissue microarray fabrication; SL Doong provided the HBx expression vector materials and offered valuable insights on the HBx-related research; YC Wu, YC Kuo, MHT Ngo, and HF Wang conducted the animal experiments; YC Wu, YH Huang, and TS Chang wrote the paper. All authors discussed the results and commented on the manuscript.

## Data availability

The datasets analyzed during the current study are publicly available in the Gene Expression Omnibus (GEO) under accession no. GSE76427.


## Acknowledgements

We thank Dr. Shin-Lian Doong (National Taiwan University, Taiwan) for providing the HBx expression vector, and Dr. Muh-Hwa Yang (National Yang Ming Chiao Tung University, Taiwan) for providing Mahlavu cells. The authors also acknowledge the Laboratory Animal Center and the Office of Research and Development at Taipei Medical University for their excellent technical support, and the service provided by the Precious Instrumentation Core Laboratory, Chang Gung Memorial Hospital, Chiayi, Taiwan.


## Supplementary data

Supplementary data to this article can be found online at https://doi.org/10.1016/j.jhepr.2025.101620.

## References

*Author names in bold designate shared co-first authorship*


[1] Bray F, Laversanne M, Sung H, et al. Global cancer statistics 2022: GLOBOCAN estimates of incidence and mortality worldwide for 36 cancers in 185 countries. CA Cancer J Clin 2024;74(3):229–263.

[2] Llovet JM, Kelley RK, Villanueva A, et al. Hepatocellular carcinoma. Nat Rev Dis Primers 2021;7(1):6.

[3] Reig M, Forner A, Rimola J, et al. BCLC strategy for prognosis prediction and treatment recommendation: the 2022 update. J Hepatol 2022;76(3):681–693.

[4] Fan ST, Poon RT, Yeung C, et al. Outcome after partial hepatectomy for hepatocellular cancer within the Milan criteria. Br J Surg 2011;98(9):1292–1300.

[5] Feng J, He Y, Wan J, et al. Pulmonary metastases in newly diagnosed hepatocellular carcinoma: a population-based retrospective study. HPB (Oxford) 2020;22(9):1295–1304.

[6] Nevola R, Ruocco R, Criscuolo L, et al. Predictors of early and late hepatocellular carcinoma recurrence. World J Gastroenterol 2023;29(8):1243–1260.

[7] **Chang TS**, **Wu YC**, Chi CC, et al. Activation of IL6/IGFIR confers poor prognosis of HBV-related hepatocellular carcinoma through

induction of OCT4/NANOG expression. Clin Cancer Res 2015;21 (1):201–210.
[8] Leupin O, Bontron S, Schaeffer C, et al. Hepatitis B virus X protein stimulates viral genome replication via a DDB1-dependent pathway distinct from that leading to cell death. J Virol 2005;79(7):4238–4245.
[9] Slagle BL, Lee TH, Medina D, et al. Increased sensitivity to the hepatocarcinogen diethylnitrosamine in transgenic mice carrying the hepatitis B virus X gene. Mol Carcinog 1996;15(4):261–269.
[10] Arzumanyan A, Friedman T, Ng IO, et al. Does the hepatitis B antigen HBx promote the appearance of liver cancer stem cells? Cancer Res 2011;71 (10):3701–3708.
[11] Wu YC, Ling TY, Lu SH, et al. Chemotherapeutic sensitivity of testicular germ cell tumors under hypoxic conditions is negatively regulated by SENP1-controlled sumoylation of OCT4. Cancer Res 2012;72 (19):4963–4973.
[12] Shinoda K, Kuboki S, Shimizu H, et al. Pin1 facilitates NF-kappaB activation and promotes tumour progression in human hepatocellular carcinoma. Br J Cancer 2015;113(9):1323–1331.
[13] Lei S, Luo M, Wang Y. Pin1 as a central node in oncogenic signaling: mechanistic insights and clinical prospects (Review). Mol Med Rep 2025;31(3).
[14] Pang R, Lee TK, Poon RT, et al. Pin1 interacts with a specific serine-proline motif of hepatitis B virus X-protein to enhance hepatocarcinogenesis. Gastroenterology 2007;132(3):1088–1103.
[15] Zheng M, Xu H, Liao XH, et al. Inhibition of the prolyl isomerase Pin1 enhances the ability of sorafenib to induce cell death and inhibit tumor growth in hepatocellular carcinoma. Oncotarget 2017;8 (18):29771–29784.
[16] Chen CH, Chang CC, Lee TH, et al. SENP1 deSUMOylates and regulates Pin1 protein activity and cellular function. Cancer Res 2013;73 (13):3951–3962.
[17] Grinchuk OV, Yenamandra SP, Iyer R, et al. Tumor-adjacent tissue co-expression profile analysis reveals pro-oncogenic ribosomal gene signature for prognosis of resectable hepatocellular carcinoma. Mol Oncol 2018;12(1):89–113.
[18] Ma S, Chan KW, Hu L, et al. Identification and characterization of tumorigenic liver cancer stem/progenitor cells. Gastroenterology 2007;132 (7):2542–2556.
[19] Zhang Q, Han Z, Zhu Y, et al. The role and specific mechanism of OCT4 in cancer stem cells: a review. Int J Stem Cells 2020;13(3):312–325.
[20] Liang C, Xu Y, Ge H, et al. Clinicopathological and prognostic significance of OCT4 in patients with hepatocellular carcinoma: a meta-analysis. Onco Targets Ther 2018;11:47–57.
[21] Zhang HJ, Siu MK, Wong ES, et al. Oct4 is epigenetically regulated by methylation in normal placenta and gestational trophoblastic disease. Placenta 2008;29(6):549–554.
[22] Zhao HX, Li Y, Jin HF, et al. Rapid and efficient reprogramming of human amnion-derived cells into pluripotency by three factors OCT4/SOX2/NANOG. Differentiation 2010;80(2–3):123–129.
[23] Lopez-Bertoni H, Lal B, Li A, et al. DNMT-dependent suppression of microRNA regulates the induction of GBM tumor-propagating phenotype by Oct4 and Sox2. Oncogene 2015;34(30):3994–4004.
[24] Lai SC, Su YT, Chi CC, et al. DNMT3b/OCT4 expression confers sorafenib resistance and poor prognosis of hepatocellular carcinoma through IL-6/STAT3 regulation. J Exp Clin Cancer Res 2019;38(1):474.
[25] Sartorius K, An P, Winkler C, et al. The epigenetic modulation of cancer and immune pathways in hepatitis B virus-associated hepatocellular carcinoma: the influence of HBx and miRNA dysregulation. Front Immunol 2021;12:661204.
[26] Bandopadhyay M, Banerjee A, Sarkar N, et al. Tumor suppressor micro RNA miR-145 and onco micro RNAs miR-21 and miR-222 expressions are differentially modulated by hepatitis B virus X protein in malignant hepatocytes. BMC Cancer 2014;14:721.
[27] Xu N, Papagiannakopoulos T, Pan G, et al. MicroRNA-145 regulates OCT4, SOX2, and KLF4 and represses pluripotency in human embryonic stem cells. Cell 2009;137(4):647–658.
[28] Wang C, Tao W, Ni S, et al. Tumor-suppressive microRNA-145 induces growth arrest by targeting SENP1 in human prostate cancer cells. Cancer Sci 2015;106(4):375–382.
[29] Agustiningsih A, Rasyak MR, Turyadi, et al. The oncogenic role of hepatitis B virus X gene in hepatocarcinogenesis: recent updates. Explor Target Antitumor Ther 2024;5(1):120–134.
[30] Wang C, Wang MD, Cheng P, et al. Hepatitis B virus X protein promotes the stem-like properties of OV6(+) cancer cells in hepatocellular carcinoma. Cell Death Dis 2017;8(1):e2560.
[31] Sanal MG, Gupta S, Saha R, et al. Hepatitis B virus X protein increases cellular OCT3/4 and MYC and facilitates cellular reprogramming. Cell Reprogram 2023;25(5):224–237.
[32] Rawal P, Tripathi DM, Hemati H, et al. Targeted HBx gene editing by CRISPR/Cas9 system effectively reduces epithelial to mesenchymal transition and HBV replication in hepatoma cells. Liver Int 2024;44(2):614–624.
[33] Lin M, Zhang M, Yi B, et al. Emerging role of SENP1 in tumorigenesis and cancer therapy. Front Pharmacol 2024;15:1354323.
[34] Jayachandran A, Dhungel B, Steel JC. Epithelial-to-mesenchymal plasticity of cancer stem cells: therapeutic targets in hepatocellular carcinoma. J Hematol Oncol 2016;9(1):74.
[35] Cui CP, Wong CC, Kai AK, et al. SENP1 promotes hypoxia-induced cancer stemness by HIF-1alpha deSUMOylation and SENP1/HIF-1alpha positive feedback loop. Gut 2017;66(12):2149–2159.
[36] Zhang W, Sun H, Shi X, et al. SENP1 regulates hepatocyte growth factor-induced migration and epithelial-mesenchymal transition of hepatocellular carcinoma. Tumour Biol 2016;37(6):7741–7748.
[37] Chang JH, Au HK, Lee WC, et al. Expression of the pluripotent transcription factor OCT4 promotes cell migration in endometriosis. Fertil Steril 2013;99 (5):1332–9 e5.
[38] Chang TS, Chen CL, Wu YC, et al. Inflammation promotes expression of stemness-related properties in HBV-related hepatocellular carcinoma. PLoS One 2016;11(2):e0149897.
[39] Cheng CW, Tse E. Targeting PIN1 as a therapeutic approach for hepatocellular carcinoma. Front Cell Dev Biol 2019;7:369.
[40] Cheng CW, Leong KW, Tse E. Understanding the role of PIN1 in hepatocellular carcinoma. World J Gastroenterol 2016;22(45):9921–9932.
[41] Mendez-Blanco C, Fondevila F, Garcia-Palomo A, et al. Sorafenib resistance in hepatocarcinoma: role of hypoxia-inducible factors. Exp Mol Med 2018;50(10):1–9.
[42] Llovet JM, Ricci S, Mazzaferro V, et al. Sorafenib in advanced hepatocellular carcinoma. N Engl J Med 2008;359(4):378–390.
[43] Cheng AL, Kang YK, Chen Z, et al. Efficacy and safety of sorafenib in patients in the Asia-Pacific region with advanced hepatocellular carcinoma: a phase III randomised, double-blind, placebo-controlled trial. Lancet Oncol 2009;10(1):25–34.
[44] Cheng Z, Wei-Qi J, Jin D. New insights on sorafenib resistance in liver cancer with correlation of individualized therapy. Biochim Biophys Acta Rev Cancer 2020;1874(1):188382.

Keywords: HBV-HCC; HBx; SENP1; CSC-associated properties.

*Received 30 November 2024; received in revised form 24 September 2025; accepted 29 September 2025; Available online 8 October 2025*

Journal of Hepatology, Volume *8*

# Supplemental information

# HBV X protein regulates cancer stemness and tumor invasiveness through SENP1 in hepatocellular carcinoma

**Yu-Chih Wu, Yen-Chiao Huang, Yung-Che Kuo, Mai-Huong Thi Ngo, Kam-Fai Lee, Yen-Tseng Sung, Hsiao-Feng Wang, Shin-Lian Doong, Liang-Mou Kuo, Te-Sheng Chang, and Yen-Hua Huang**

# HBV X protein regulates cancer stemness and tumor invasiveness through SENP1 in hepatocellular carcinoma

Yu-Chih Wu, Yen-Chiao Huang, Yung-Che Kuo, Mai-Huong Thi Ngo, Kam-Fai Lee, Yen-Tseng Sung, Hsiao-Feng Wang, Shin-Lian Doong, Liang-Mou Kuo, Te-Sheng Chang, Yen-Hua Huang

Table of contents

Supplementary materials and methods........................................................................2

Supplementary tables..........................................................................................10

Supplementary figures.........................................................................................13

Supplementary references....................................................................................21

## Supplementary materials and methods

### *Plasmid construction, short hairpin RNA, and lentiviral transduction*

The HBx-IRES-GFP lentiviral vector, which encodes the full-length HBx protein, was reconstructed from the pRT-HBx-GFP plasmid, which was kindly provided by Dr. Shin-Lian Doong (1). The HA-SENP1-IRES-tRFP lentiviral vector was generated in a previous study (2). The LentimiRa-hsa-miR-145-5p Vector (Cat. No. mh40185) for miR-145 overexpression was purchased from Applied Biological Materials Inc. in Canada. The plasmids encoding short hairpin RNAs for SENP1 (shSENP1#1, TRCN0000004395; shSENP1#2, TRCN0000004396), OCT4 (shOCT4#1, TRCN00004879; shOCT4#2, TRCN00004881), and shControl. (shCtrl., TRCN0000072224) were purchased from the National RNAi Core Facility at Academia Sinica in Taiwan. For viral production, the packaging pCMVΔR8.91 plasmid and the envelope VSV-G pMD.G plasmid were cotransfected with the shRNA plasmids into HEK293T cells by using the Turbofect transfection reagent according to the manufacturer's instructions (Thermo Fisher Scientific, USA). After 12-h transfection, the cells were washed with phosphate-buffered saline (PBS), and the cells were then cultured in 10 mL of medium containing 10% FBS. The virus particles were collected at 48 and 72 h post-transfection, filtered through 0.45-μm filters, and concentrated by ultracentrifugation at 140,000 ×*fg* at 4°C for 2.5 h. The cells were infected with the lentivirus in the presence of 8 μg/mL polybrene (Sigma-Aldrich, USA).

### *Database analysis*

For data mining, the gene expression data of the peri-tumor and tumor tissues were downloaded from the Gene Expression Omnibus (GEO) website (GSE76427) (3). The gene set enrichment algorithm (GSEA) was implemented using MSigDB. Moreover, 115 patients with HCC from the GSE76427 dataset were divided into two groups based on their median SENP1 expression level (i.e., SENP1_High and SENP1_Low). Then, the raw data from the GSE76427 dataset were analyzed through GSEA in

reference to the HCC recurrence signature (WOO_LIVER_CANCER_RECURRENCE_UP [M12602] and WOO_LIVER_CANCER_RECURRENCE_DN [M9911]) and embryonic stem cell (ESC) signature (WONG_EMBRYONIC_STEM_CELL_CORE [M7079]) gene sets (3-5). The WOO_LIVER_CANCER_RECURRENCE_UP and WOO_LIVER_CANCER_RECURRENCE_DN gene sets contain the genes positively and negatively correlated with HCC recurrence. The WONG_EMBRYONIC_STEM_CELL_CORE gene set contains the genes coordinately upregulated in a compendium of human ESCs.

***RNA isolation and real-time quantitative polymerase chain reaction***

Total RNA was isolated from the cell lines and frozen HCC tissues and was subjected to real-time quantitative polymerase chain reaction (qPCR). Specifically, for the cell lines, total RNA was extracted using the RNeasy Micro Kit (Qiagen, USA) according to the manufacturer’s instructions. Frozen tissues were homogenized in liquid $N_2$ and lysed in RNA extraction buffer. Moreover, 3 μg of total RNA was used to synthesize complementary DNA (cDNA) with a random primer (Thermo Fisher Scientific), and in a final volume of 20 μL, cDNA synthesis was performed using Superscript III reverse transcriptase (Thermo Fisher Scientific) at 42°C for 50 min according to the manufacturer’s instructions. For qPCR amplification, the Fast SYBR Green Master Mix (Thermo Fisher Scientific) was utilized. The primers used in the study are listed in Table S1. Specifically, for the quantification of miR-145 expression, small RNA was extracted from HepG2 cells using the NucleoSpin miRNA kit (Takara Bio, Japan). The cDNA synthesis was conducted using the Mir-X miRNA First Strand Synthesis kit (Takara Bio), adhering to the manufacturer’s guidelines. Subsequent qPCR was performed using the Mir-X™ miRNA qRT-PCR TB Green kit (Takara Bio) with a miR-145-specific primer (5’-GTCCAGTTTTCCCAGGAATCCCT-3’) and the mRQ 3' universal primer. Each reaction was conducted in triplicate, and miR-145 expression levels were normalized to U6 snRNA using the ΔΔCt method.

***Western blot analysis***

Total protein was extracted using RIPA lysis buffer supplemented with a protease inhibitor cocktail (Roche Diagnostics). Protein concentration was determined using a BCA protein quantification kit (Pierce, USA). For the Western blot analysis, 20 μg of total protein was denatured by boiling in Laemmli buffer, separated by 10% sodium dodecyl sulfate-polyacrylamide gel electrophoresis (SDS-PAGE), and subsequently transferred to a polyvinylidene difluoride (PVDF) membrane. The PVDF membranes were blocked with 5% skim milk in Tris-buffered saline containing 0.05% Tween-20 (TBST) for 1 hour at room temperature. Membranes were then incubated with primary antibodies overnight at 4°C. A detailed list of primary antibodies used is provided in Table S2. Following primary antibody incubation, the membranes were incubated with horseradish peroxidase (HRP)-conjugated goat anti-rabbit or rabbit anti-mouse IgG secondary antibodies for 1 hour at room temperature. After four washes with TBST, immunoreactive bands were visualized using the Immobilon Western blotting kit (EMD Millipore, Germany).

***Tissue microarray development and immunohistochemical staining***

Matched pairs of paraffin-embedded primary HCC tissue samples and adjacent liver tissues were used for the construction of a tissue microarray (TMA). Briefly, to obtain representative tissue regions, hematoxylin and eosin-stained sections were prepared from each selected donor block. To produce the TMA block, tissue cylinders (1.5 mm in diameter) were punched from the donor block and were transferred to an 18 × 30-mm paraffin block by using an automatic tissue microarrayer (AutoTiss 1000, EverBio Technology, Canada). The resulting TMA block was cut into 5-μm sections, which were placed on 3-aminopropyltriethoxysilane-coated glass slides by using a sliding microtome (Leica SM 2000 R, Meyer Instruments, USA). Immunohistochemical staining was performed on sections obtained from TMA and mouse tissues. Following deparaffinization and rehydration, the slides were washed and blocked with 5% normal horse serum in phosphate-buffered saline (PBS) to reduce non-specific binding. The slides were then incubated with the primary antibodies overnight at 4°C. A list

of the primary antibodies used is provided in Table S2. After incubation, the slides were treated with a horseradish peroxidase (HRP)-conjugated secondary antibody. The intensity of the positive signals was visualized using TissueFAXS imaging system and analyzed using TissueQuest analysis software (TissueGnostics).

***Immunocytochemistry staining***

Cells were fixed in 4% paraformaldehyde at room temperature for 30 min. After fixation, cells were rinsed two times with PBS at room temperature for 30 min and blocked with bovine serum albumin (BSA; 50 mg/mL) and 0.5% triton X-100 in PBS for 1 h at room temperature. Cells were then incubated with the anti-SENP1 antibody (sc-271360, Santa Cruz Biotechnology) or anti-PIN1 antibody (sc-46660, Santa Cruz Biotechnology), and labeling with the primary antibody was detected using Cy3-conjugated secondary antibodies (Jackson ImmunoResearch, Bar Harbor, ME, USA). The nuclei of all cells were counterstained with 4',6-diamidino-2-phenylindole (DAPI, Sigma-Aldrich). All cells were covered with an anti-fading reagent (Vector Laboratories, Burlingame, CA, USA) and were analyzed under a fluorescence microscope.

***Flow cytometry analysis***

HepG2 cells, GFP-HepG2 cells and HBx-GFP-HepG2 cells were harvested, washed twice with phosphate-buffered saline (PBS), and resuspended in PBS containing 1% bovine serum albumin (BSA). The cells were then incubated with the PE-conjugated anti-CD133 antibody (BD bioscience, USA) for 30 minutes at 4°C in the dark. Following incubation, the cells were washed twice with PBS to remove unbound antibody. Flow cytometry analysis was performed using the BD FACSVerse flow cytometer. Data acquisition and analysis were conducted using BD FACSDiva software (BD Biosciences).

***Wound-closure and transwell migration assays***

For the wound-closure assay, cells were added to 60-mm plates at equivalent cell densities. A

micropipette tip was used to scratch a wound in each cell monolayer. The plates were washed with medium to remove the detached cells. Subsequently, the adherent cells were incubated in 5% $CO_2$ humidified atmosphere at 37°C for 12-24 h. Digital images of the scratch-wound area were acquired at each time point, and the area of the gap was measured. For the transwell assay, cells ($2 \times 10^5$) were seeded into a Matrigel-coated transwell insert (8-μm pore size, Corning Costar, USA) for 24 h. Uninvaded cells were removed with a cotton swab, and cells that had passed through the lower membrane of the transwell insert were fixed, stained with a crystal violet solution, and observed under an inverted microscope.

***Tumor sphere formation assay***

Cells ($2 \times 10^3$) were seeded into a 6-well ultra-low attachment plate (Corning Costar), and cells were maintained in serum-free DMEM-F12 supplemented with 20 ng/mL epidermal growth factor (EGF; PeproTech, USA), 25 ng/mL basic fibroblast growth factor (bFGF; PeproTech), and B27 (Thermo Fisher Scientific) for 14 days for primary tumor sphere formation. The primary spheres were dissociated after 7 days, gently trypsinized, counted and then re-seeded for secondary tumor sphere formation. Tumor spheres were observed and counted under a light microscope. Secondary tumor spheres were observed under a light microscope, and the number of spheres with a diameter greater than 100 μm was counted. The experiment was conducted in triplicate, and the average number of tumor spheres was calculated across the replicates.

***Dual luciferase assay***

The SENP1-promoter luciferase plasmids, in which the SENP1 promoter was fused with firefly luciferase reporter gene, were cotransfected with the control Renilla luciferase plasmid (pRL-TK) into HepG2 cells (6). Following cotransfection, HepG2 cells were lysed using the cell lysis buffer provided with the Dual Luciferase Reporter 1000 Assay System (Promega, USA), and relative luciferase activity was assessed using a luminometer, Relative luciferase activity is used to represent the ratio of firefly

luciferase activity to Renilla luciferase activity.

***Targeting 3'UTR luciferase reporter assay***

The SENP1 3'- untranslated region (UTR) sequence, which contains a predicted miR-145-5p binding site, was cloned into a pMirTarget 3'-UTR assay vector (Origene Technologies, Inc., USA) following digestion with the restriction enzymes EcoRI and XbaI. HepG2 cells were infected with the LentimiRa-hsa-miR-145-5p lentivirus, and subsequently transfected with the pMirTarget SENP1 3'-UTR luciferase reporter constructs using Lipofectamine™ 3000 transfection reagent (Thermo Fisher Scientific). After 48 hours of incubation, firefly luciferase activity was measured using the Luciferase Reporter Assay System (Promega). The luciferase activity data were normalized to RFP signals and subsequently analyzed with the SpectraMax iD5 Multi-Mode Microplate Reader (Molecular Devices, USA).

***Generation of reporter gene cell line***

HepG2 cells stably expressing reporter genes were generated by lentiviral vector transduction. The following pLAS3w. Fluc.Ppuro lentiviral vectors from the National RNAi Core Facility at Academia Sinica in Taiwan were used. Cells were infected with lentivirus to express firefly luciferase and carry the puromycin resistance gene, followed by puromycin selection to establish a stable reporter cell line. Using this stable reporter cell line, we overexpressed HBx or silenced SENP1, followed by orthotopic xenograft mouse experiments

***Orthotopic xenograft mouse model and* sorafenib treatment *in vivo***

Eight-week-old immunodeficient (Nu/Nu) mice were obtained from the National Laboratory Animal Center, Taiwan. Each mouse was anesthetized, and through an transverse incision made in the upper abdomen by using a micro-syringe, each mouse was orthotopically inoculated in the left hepatic lobe. For tumor growth monitoring, tumors were imaged by IVIS Lumina III XRMS (PerkinElmer Inc.,

USA). Before imaging, 100 µl of 30 mg/mL D-luciferin (Bioxynth, UK) dissolved in DPBS was intraperitoneally injected into each mouse, and 15 min later, the mice were anesthetized with isoflurane and imaged by the IVIS imaging system. After 8 weeks, the mice were sacrificed, and their livers and lungs were dissected, fixed with phosphate-buffered neutral formalin, and prepared for standard histological examination. The number of lung metastatic tumors in mice was determined using tissue sections stained with hematoxylin and eosin (HE). These sections were observed under a microscope, and the quantity of lung metastatic tumor area was counted. To evaluate the therapeutic response to sorafenib, mice in four experimental groups (GFP-shCtrl., GFP-shSENP1, HBx-shCtrl., HBx-shSENP1) were administered sorafenib intraperitoneally at a dose of 30 mg/kg, twice per week, beginning at week 8 post-cell inoculation. Tumor progression was monitored weekly using bioluminescence imaging (IVIS Lumina III XRMS, PerkinElmer Inc., USA). Bioluminescence signals (photons/second) obtained at week 8 served as the baseline and were normalized to 100% for subsequent longitudinal comparisons. The animal study protocol was approved by the Institutional Animal Care and Use Committee at Taipei Medical University, Taipei, Taiwan (Approval number: LAC-2017-0022, LAC-2025-0040).

### *Dose-response profiles of sorafenib in HCC cell lines*

Cells were seeded at a density of 5,000 cells per well in 96-well plates and treated with sorafenib (Cell Signaling Technology, USA), SENP1-IN-3 (MedChemExpress, USA), or their combination for 48 hours. Cell viability was assessed using the WST-1 assay.

### *Statistical analysis*

Data are presented as mean ± standard deviation (SD), as appropriate. The statistical differences in the means were assessed using the paired Student's *t* test, Mann-Whitney test, or Spearman's correlation analysis. Kaplan-Meier analysis was used to examine overall survival (OS) and disease-free survival (DFS), and the Peto-Prentice test was used to determine OS and DFS. $P < 0.05$ indicated statistical

significance. GraphPad Prism 9 software for Windows was used for statistical analysis.

# Supplementary tables

## Table S1. List of real-time quantitative PCR primers

| Gene | Accession | Forward Primers | Reverse Primers |
|---|---|---|---|
| SENP1 | NM_001267594 | 5’-TGGCCAGAGTGCAAATGG-3’ | 5’-TCGGCTGTTTCTTGATTTTTGTAA-3’ |
| OCT4 | NM_002701 | 5’-CAACTCCGATGGGGCCT-3’ | 5’-CTTCAGGAGCTTGGCAAATTG-3’ |
| SNAIL | NM_001078353 | 5’-CTTCCAGCAGCCCTACGAC-3’ | 5’-CGGTGGGGTTGAGGATCT-3’ |
| TWIST | NM_001165012 | 5′-TCTCGGTCTGGAGGATGGAG-3′ | 5′-GTTATCCAGCTCCAGAGTCT-3′ |
| β-2M | NM_004048 | 5’-GATGAGTATGCCTGCCGTGTG-3’ | 5’-CAATCCAAATGCGGCATCT-3’ |

## Table S2. List of antibodies

| Protein | Assay | Cat. No. | Supplier | Origin | Dilution | Incubation Period |
|---|---|---|---|---|---|---|
| SENP1 | IHC | NBP1-89553 | Novus | rabbit | 1:100 | overnight, 4°C |
| OCT4 | IHC | sc-5279 | Santa Cruz Biotech | mouse | 1:100 | overnight, 4°C |
| CD133 | IHC | ab19898 | Abcam | rabbit | 1:200 | overnight, 4°C |
| N-Cadherin | IHC | 610920 | BD bioscience | mouse | 1:200 | overnight, 4°C |
| E-Cadherin | IHC | 610182 | BD bioscience | mouse | 1:200 | overnight, 4°C |
| PIN1 | IHC | sc-15340 | Santa Cruz Biotech | rabbit | 1:100 | overnight, 4°C |
| SENP1 | WB | sc-271360 | Santa Cruz Biotech | mouse | 1:1000 | overnight, 4°C |
| OCT4 | WB | sc-5279 | Santa Cruz Biotech | mouse | 1:500 | overnight, 4°C |
| N-Cadherin | WB | 610920 | BD bioscience | mouse | 1:1000 | overnight, 4°C |
| E-Cadherin | WB | 610182 | BD bioscience | mouse | 1:1000 | overnight, 4°C |
| SNAIL | WB | GTX125918 | GeneTex | rabbit | 1:1000 | overnight, 4°C |
| TWIST | WB | GTX127310 | GeneTex | rabbit | 1:1000 | overnight, 4°C |
| HBx | WB | sc-71239 | Santa Cruz Biotech | mouse | 1:1000 | overnight, 4°C |
| CD133 | WB | #64326 | Cell Signaling | rabbit | 1:2000 | overnight, 4°C |
| PIN1 | WB | sc-15340 | Santa Cruz Biotech | mouse | 1:2000 | overnight, 4°C |
| CyclinD1 | WB | 2261-1 | Epitomic | rabbit | 1:20000 | overnight, 4°C |
| p-STAT3 | WB | #9145 | Cell Signaling | rabbit | 1:2000 | overnight, 4°C |
| STAT3 | WB | #9132 | Cell Signaling | rabbit | 1:2000 | overnight, 4°C |
| IGF-1β | WB | sc-713 | Santa Cruz Biotech | rabbit | 1:1000 | overnight, 4°C |
| β-ACTIN | WB | A5441 | Sigma-Aldrich | mouse | 1:10000 | overnight, 4°C |
| CD133 | FC | 566593 | BD bioscience | mouse | 1:100 | 30 minutes, 4°C |
| SENP1 | ICC | NBP1-89553 | Novus | rabbit | 1:100 | overnight, 4°C |
| PIN1 | ICC | sc-15340 | Santa Cruz Biotech | rabbit | 1:100 | overnight, 4°C |

IHC, immunohistochemistry; WB, Western blot; FC, flow cytometry;
ICC, immunocytochemistry

**Table.S3 Number of primary tumors, intrahepatic metastasis and pulmonary metastasis in orthotopic xenograft mice.**

| Group | Exp. Condition | | Primary tumor | Intrahepatic metastasis | Pulmonary metastasis |
|---|---|---|---|---|---|
| **1** | GFP | shCtrl. | 3/4 | 1/4 | 1/4 |
| **2** | | shSENP1 | 1/4 | 0/4 | 0/4 |
| **3** | HBx | shCtrl. | 4/4 | 4/4 | 3/4 |
| **4** | | shSENP1 | 2/3 | 1/3 | 0/3 |

## Supplementary figures

### Fig. S1

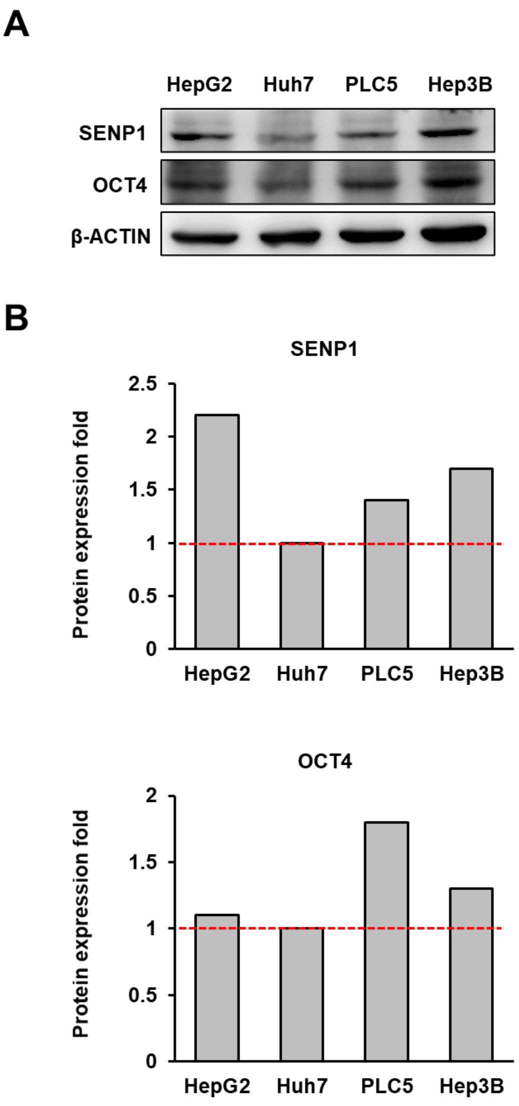


**Fig. S1. SENP1 and OCT4 protein levels in various commercialized HCC cell lines.**

**A,** SENP1 and OCT4 protein levels in in various cells were analyzed through Western blotting. **B,** Quantitative data are shown in terms of protein levels.

**Fig. S2**

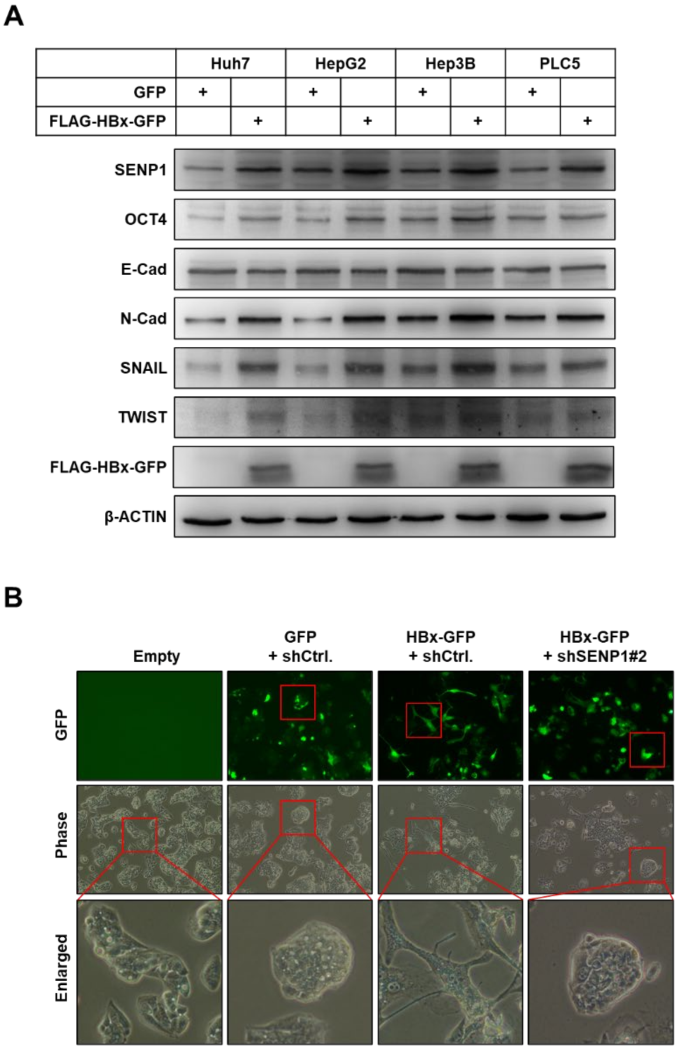


**Fig. S2. HBx increases CSC and EMT-associated factors in HCC.**

**A,** Huh7 and HepG2 ($HBV^-$ cell line) and Hep3B and PLC5 ($HBV^+$ cell lines) were infected the lentivirus carrying the HBx-GFP plasmid or GFP control vector. Relative SENP1, OCT4, E-Cadherin, N-Cadherin, SNAIL, TWIST, and HBx protein levels were examined through Western blotting. **B,** HBx-induced mesenchymal-like cell morphology was changed into epithelial-like cell morphology through SENP1 silencing.

**Fig. S3**

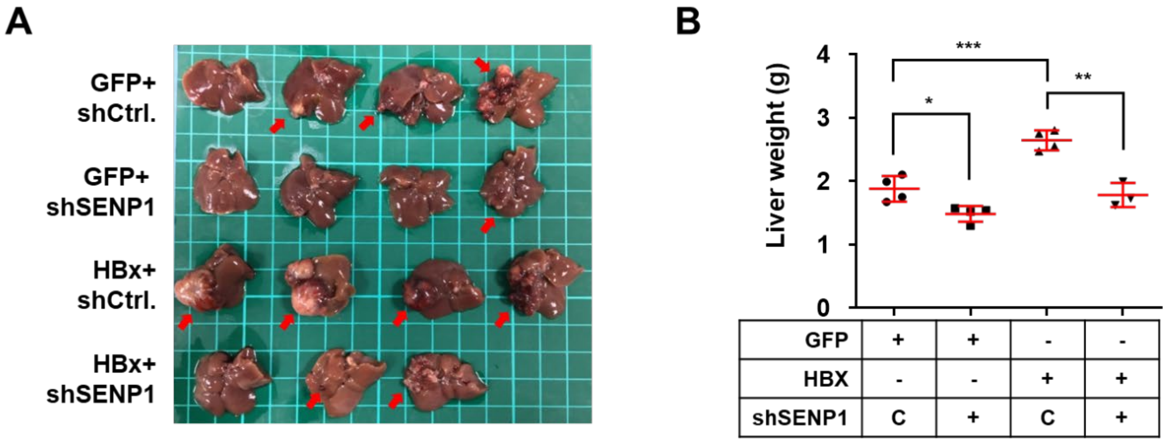


**Fig. S3. SENP1 knockdown reduces HBx-induced liver enlargement in orthotopic xenograft model.**

**A,** Representative liver tumor images from four groups of mice: GFP-shCtrl., GFP-shSENP1, HBx-shCtrl., and HBx-shSENP1. Red arrows indicate tumor samples selected for histological analysis, which are presented as representative images in Fig. 6H. **B,** Quantification of liver weights in the indicated groups. *$P < 0.05$, **$P < 0.01$, ***$P < 0.001$, Mann-Whitney test.

**Fig. S4**

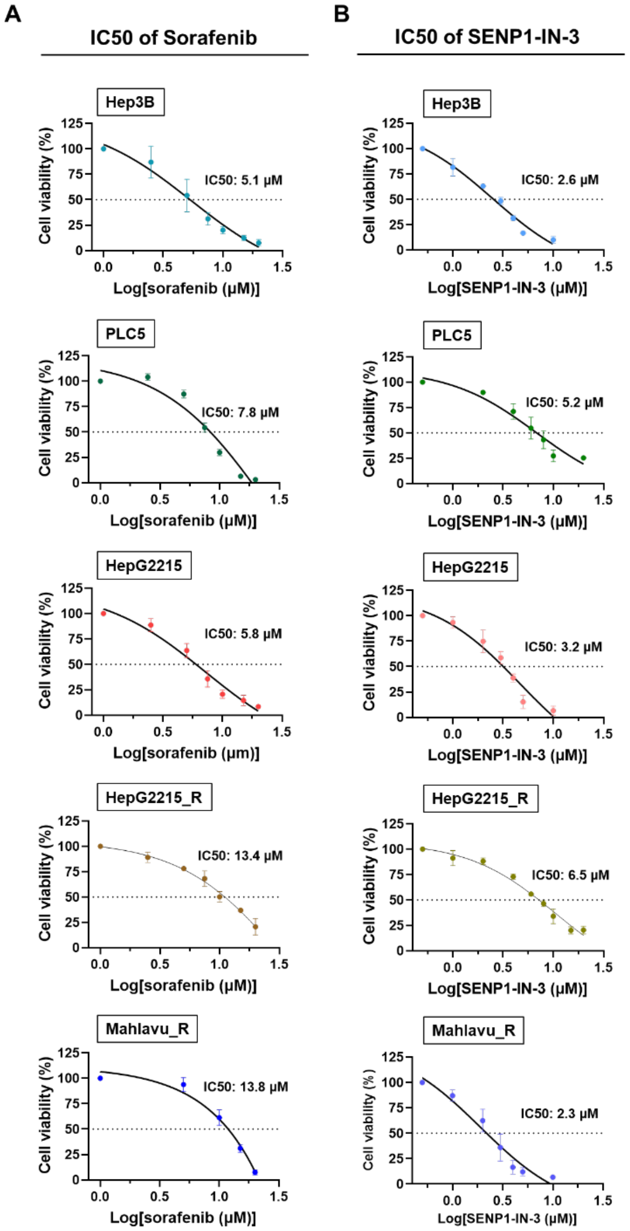


**Fig. S4. Dose-response analysis of sorafenib and SENP1-IN-3 in HCC cell lines.**

**A,** IC50 values of sorafenib. **B,** IC50 values of SENP1-IN-3.

Naïve cells (Hep3B, PLC5, and HepG2215) and sorafenib-resistant cells (HepG2215_R and Mahlavu_R) HCC cell lines were treated with increasing concentrations of sorafenib or SENP1-IN-3 for 48 hours. Cell viability was determined using the WST-1 assay. Data represent mean ± SD from more than three independent experiments.

Fig. S5

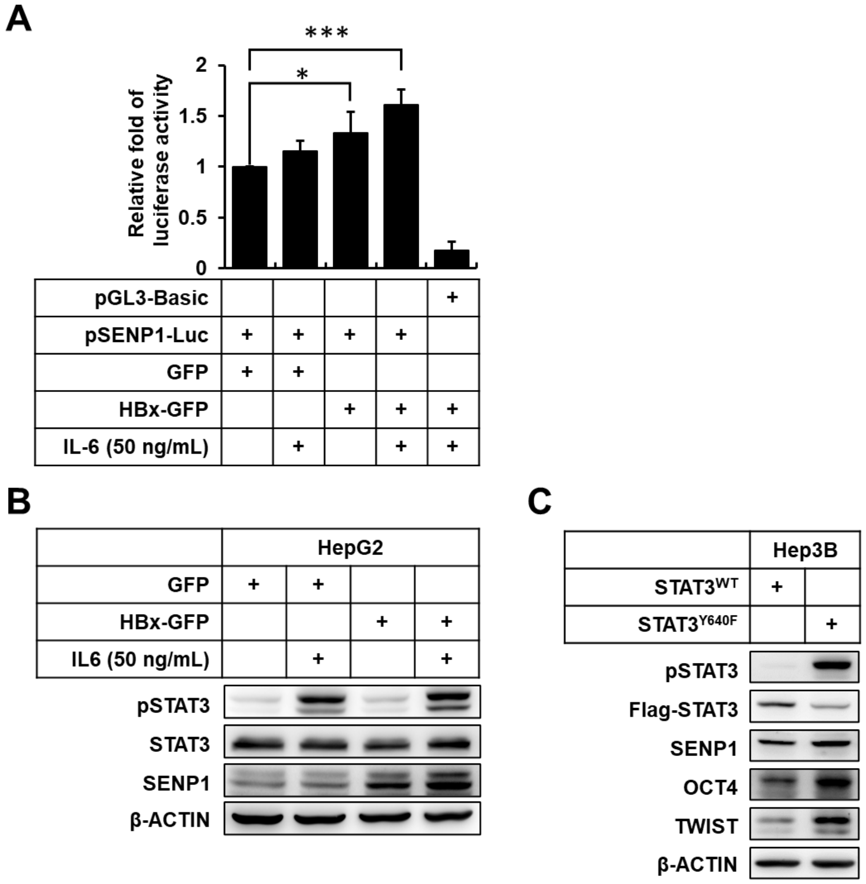


**Fig. S5. HBx/IL-6/STAT3 signaling pathway involves SENP1 and OCT4 expression in HBV-related HCC.**

**A,** SENP1 promoter luciferase reporter construct (pSENP1-Luc) or pGL3-empty reporter plasmid was cotransfected with GFP or HBx-GFP plasmids into HepG2 cells. Relative luciferase activity of SENP1 promoter-luciferase HepG2 cells with IL-6 treatment (50 ng/mL) is presented. *$P < 0.05$, ***$P < 0.001$, Student's *t* test. **B,** GFP- and HBx-GFP-HepG2 cells treated with IL-6 (50 ng/mL). Relative phosphorylated STAT3 (pSTAT3), total STAT3 (STAT3), SENP1, and HBx protein expression levels detected through Western blotting. **C,** Flag-tagged wild-type $STAT3^{WT}$ expression plasmid and a single mutant $STAT3^{Y640F}$ expression plasmid (a constitutive active STAT3) transfected into Hep3B cells. Phosphorylated STAT3 (pSTAT3), Flag-STAT3, SENP1, OCT4 and TWIST protein levels were detected through Western blotting. β-ACTIN is a loading control.

**Fig. S6**

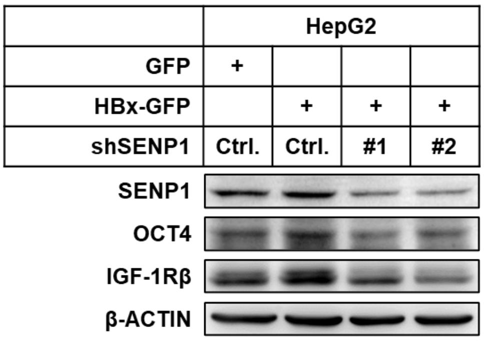


**Fig. S6. HBx-induced SENP1 involves IGF-1R and OCT4 expression in HepG2 cells.**

SENP1 expression was silenced in GFP- and HBx-GFP-HepG2 cells with shCtrl. and shSENP1 (clone#1 and #2). Relative SENP1, OCT4, IGF-1R β subunit, and β-ACTIN protein levels were examined through Western blotting.

Fig. S7

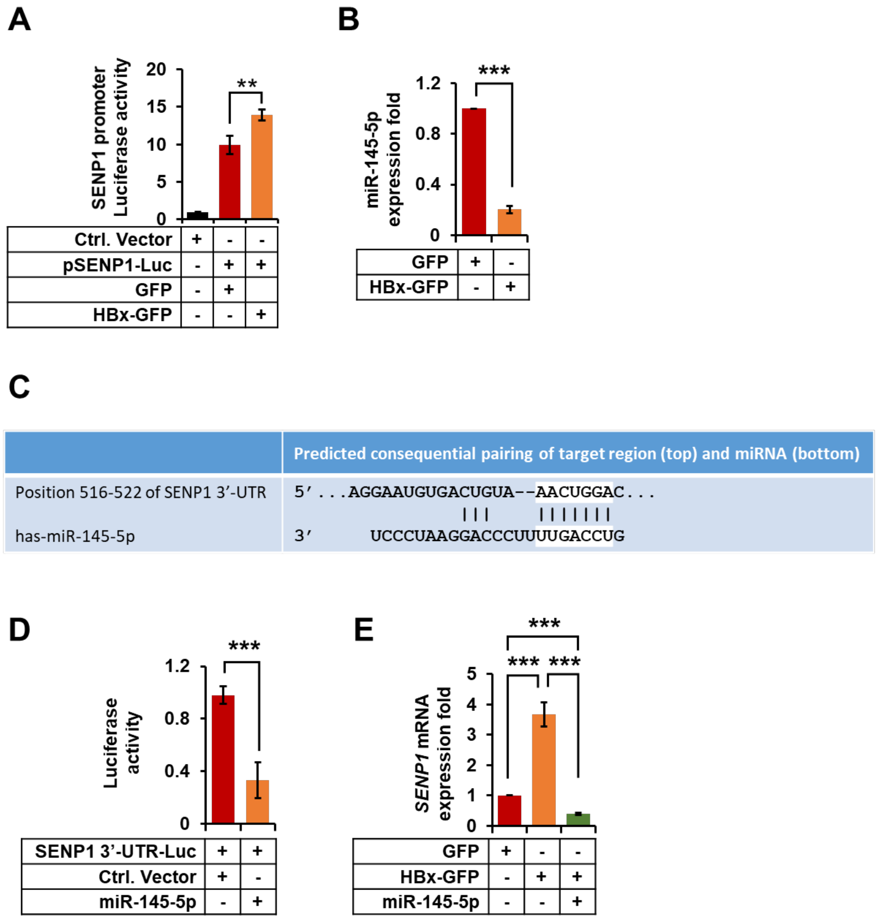


**Fig. S7. SENP1 is a direct target of miR-145-5p in HepG2 cells.**

**A,** Luciferase reporter assay of SENP1 promoter activity in HepG2 cells. **B,** MiR-145 expression levels in GFP- or HBx-overexpressing HepG2 cells were analyzed through qPCR. **C,** Sequence alignment of miR-145-5p with SENP1 3'-UTR. **D,** HepG2 cells were infected with lentivirus carrying miR-145-5p alongside pMirTarget SENP1 3ˊ-UTR luciferase reporter constructs for 48 h. Luciferase activity was measured and normalized to the RFP intensity. **E,** HepG2 cells were transduced with different expression plasmids, and *SENP1* mRNA expression levels were analyzed through qPCR. $^{**}P < 0.01$, $^{***}P < 0.001$, paired Student's *t* test.

**Fig. S8**

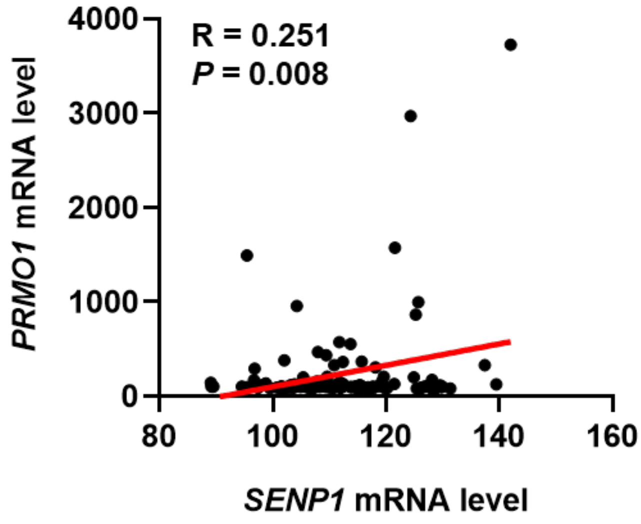


**Fig. S8. SENP1 expression positively correlates with CD133 (*PROM1*) mRNA levels in HCC tissue samples.**

A significant positive correlation between SENP1 and CD133 (*PROM1*) mRNA expression was observed in HCC specimens. The data were obtained from the GEO database (GSE76427). Pearson's correlation test.

## Supplementary references


1. Shih WL, Kuo ML, Chuang SE, Cheng AL, Doong SL. Hepatitis B virus X protein inhibits transforming growth factor-beta -induced apoptosis through the activation of phosphatidylinositol 3-kinase pathway. J Biol Chem. 2000;275(33):25858-64.
2. Wu YC, Ling TY, Lu SH, Kuo HC, Ho HN, Yeh SD, et al. Chemotherapeutic sensitivity of testicular germ cell tumors under hypoxic conditions is negatively regulated by SENP1-controlled sumoylation of OCT4. Cancer Res. 2012;72(19):4963-73.
3. Grinchuk OV, Yenamandra SP, Iyer R, Singh M, Lee HK, Lim KH, et al. Tumor-adjacent tissue co-expression profile analysis reveals pro-oncogenic ribosomal gene signature for prognosis of resectable hepatocellular carcinoma. Mol Oncol. 2018;12(1):89-113.
4. Wong DJ, Liu H, Ridky TW, Cassarino D, Segal E, Chang HY. Module map of stem cell genes guides creation of epithelial cancer stem cells. Cell Stem Cell. 2008;2(4):333-44.
5. Bhattacharya B, Miura T, Brandenberger R, Mejido J, Luo Y, Yang AX, et al. Gene expression in human embryonic stem cell lines: unique molecular signature. Blood. 2004;103(8):2956-64.
6. Wang C, Tao W, Ni S, Chen Q, Zhao Z, Ma L, et al. Tumor-suppressive microRNA-145 induces growth arrest by targeting SENP1 in human prostate cancer cells. Cancer Sci. 2015;106(4):375-82.
